# Programmed Probiotics Generate Interleukin-37b-Bearing Nanovesicles In Situ for Precision Immunomodulation in Colitis

**DOI:** 10.34133/research.1413

**Published:** 2026-08-28

**Authors:** Xiaojing Zhang, Liufei Gong, Haiyi Zhang, Yang Luo, Tengfei Long, Qian He, Yang Luo, Lu Han, Zhipeng Li, Cang Li, Xiaoyu Wang, Zijian Zheng, Yao Deng, Xiaoping Liao, Hao Ren, Jian Sun

**Affiliations:** ^1^ State Key Laboratory of Animal Disease Control and Prevention, South China Agricultural University, Guangzhou, China.; ^2^ Guangdong Provincial Key Laboratory of Veterinary Pharmaceutics Development and Safety Evaluation, South China Agricultural University, Guangzhou, China.; ^3^ National Risk Assessment Laboratory for Antimicrobial Resistance of Animal Original Bacteria, College of Veterinary Medicine, South China Agricultural University, Guangzhou, China.

## Abstract

Genetically engineered probiotic bacteria are promising living therapeutics. However, their therapeutic potential is frequently confined to their colonization sites, and limitations in efficacy, controllability, and robustness remain major barriers to translation. Anti-inflammatory cytokines play a crucial role in mitigating the pathogenesis of inflammation-related diseases, yet their application is limited by their vulnerability in the mammalian gastrointestinal tract. In this regard, we first found that interleukin-37b (IL-37b) mitigated inflammatory responses at picogram concentrations in primary intestinal cells, supporting its translational potential for the oral treatment of inflammatory diseases. Then, we harnessed the probiotic bacterium *Escherichia coli* Nissle 1917 as a chassis and further engineered it into a hypervesiculating strain, ΔECIL-37b, capable of efficiently secreting IL-37b-bearing bacterial outer membrane vesicles (OMVs) in situ. These OMVs facilitated IL-37b penetration across the intestinal barrier, enabling it to reach the inflamed mucosa and interact with target cells. In both acute and chronic murine colitis models, we demonstrated that ΔECIL-37b achieved anti-inflammatory efficacy comparable to direct IL-37b injection while enabling local intestinal delivery. Mechanistically, ΔECIL-37b exerted anti-inflammatory effects by attenuating myeloid differentiation primary response 88 (MyD88)-related downstream extracellular-signal-regulated kinase/nuclear factor κB signaling and inflammatory-immune cross-talk. Together, these findings provide a proof-of-concept strategy to harness the anti-inflammatory activity of IL-37b via probiotic-derived OMV delivery and offer insights into a precision anti-inflammatory approach integrating cytokine and probiotic functions.

## Introduction

The gastrointestinal (GI) tract hosts a complex microbial ecosystem, where probiotics regulate host microbiota homeostasis and mediate physiological functions, making them key therapeutic approaches for GI disorders [1]. In recent years, genetically engineered probiotics capable of secreting biopharmaceuticals such as cytokines and therapeutic enzymes have emerged as promising living therapeutics [2]. Their ability to colonize the intestine and generate bioactive molecules in situ offers opportunities for sustained local treatment and modulation of host–microbiota interactions [3]. Engineered probiotic immunotherapy has been reported for managing inflammatory bowel disease (IBD) [4], diabetes [5], hyperlipidemia [6], and various malignancies [7]. However, the application of this strategy to immunomodulatory biologics still requires careful evaluation. On the one hand, the amount of therapeutic protein secreted by bacteria is limited; on the other hand, the intestinal barrier prevents efficient transport of bacterial cargos to target cells in the lamina propria. These limitations highlight the need for improved probiotic-based strategies for localized immunotherapy.

Accumulating evidence indicates that bacteria can deliver bioactive molecules to host cells through the secretion of outer membrane vesicles (OMVs). As nanoscale proteoliposomal structures, OMVs can transport cargos derived from the parental bacterium and mediate bacteria–host communication [8]. In the intestinal setting, probiotic-derived OMVs have also been implicated in microbial interactions and the regulation of mucosal homeostasis [9]. Recent advances in OMV engineering and nanozyme development have further expanded their potential applications in inflammatory disease therapy and immunomodulation [10–12]. Moreover, probiotic-secreted OMVs carry microbe-associated molecular cues that can be recognized by immune cells in the lamina propria, thereby potentially facilitating the local delivery of therapeutic cargos to target cells [13]. However, their local production remains limited, bacterial carriers may be lost during intestinal delivery, and the stability and effective exposure of vesicle-associated cargos are still difficult to control. As a result, it remains unclear whether probiotic-derived OMVs can provide sufficient delivery efficiency for localized protein immunotherapy in intestinal disease.

We focused on interleukin-37b (IL-37b), an immunomodulatory cytokine uniquely expressed in humans and without a known homolog in mice or nonhuman primates. Among the 5 isoforms (IL-37a to IL-37e), IL-37b is the best characterized [14]. It functions as a natural immunosuppressive factor, showing low basal expression in healthy tissues but marked up-regulation in response to inflammatory stimuli such as lipopolysaccharide (LPS) or IL-1β, thereby contributing to a feedback inhibitory loop. Notably, previous studies have shown that IL-37b can suppress inflammatory responses at picogram concentrations in peripheral blood mononuclear cells (PBMCs) [15]. Extracellularly, IL-37b binds to IL-18 receptor α (IL-18Rα) and recruits IL-1R8 to form a complex that suppresses downstream proinflammatory signaling [16]. Intracellularly, it interacts with Smad3, thereby modulating inflammatory responses, immune activation, tissue repair, and barrier integrity [17]. These properties underscore the therapeutic potential of IL-37b in inflammatory disorders, including colitis [18]. Moreover, current engineering approaches—such as viral vectors, neutrophil-derived nanovesicles, and composite barrier materials designed for IL-37b delivery—further underscore its applicability. However, its translation remains limited by challenges in stability, targeting specificity, and efficient local delivery. Moreover, it remains unclear whether the low-dose activity observed in PBMCs can be reproduced in intestinal cells and translated into effective local therapy in the gut.

To address these limitations, an inducible probiotic system, designated ΔECIL-37b, was constructed using *Escherichia coli* Nissle 1917 (EcN) as an intestinal chassis for the in situ generation of IL-37b-bearing OMVs. This design links local bacterial production in the gut with vesicle-mediated delivery to the intestinal mucosa, while taking advantage of the potent anti-inflammatory activity of IL-37b at low doses. Furthermore, this study investigated the ability of locally generated IL-37b-bearing OMVs to alleviate colitis by modulating inflammatory-immune cross-talk and restoring epithelial barrier integrity. Together, this work provides a proof-of-concept framework for engineered probiotic-based cytokine delivery and explores the potential of local IL-37b immunomodulation in intestinal inflammatory disease.

## Results

### IL-37b is a potent anti-inflammatory modulator at picogram doses

**Fig. 1.**
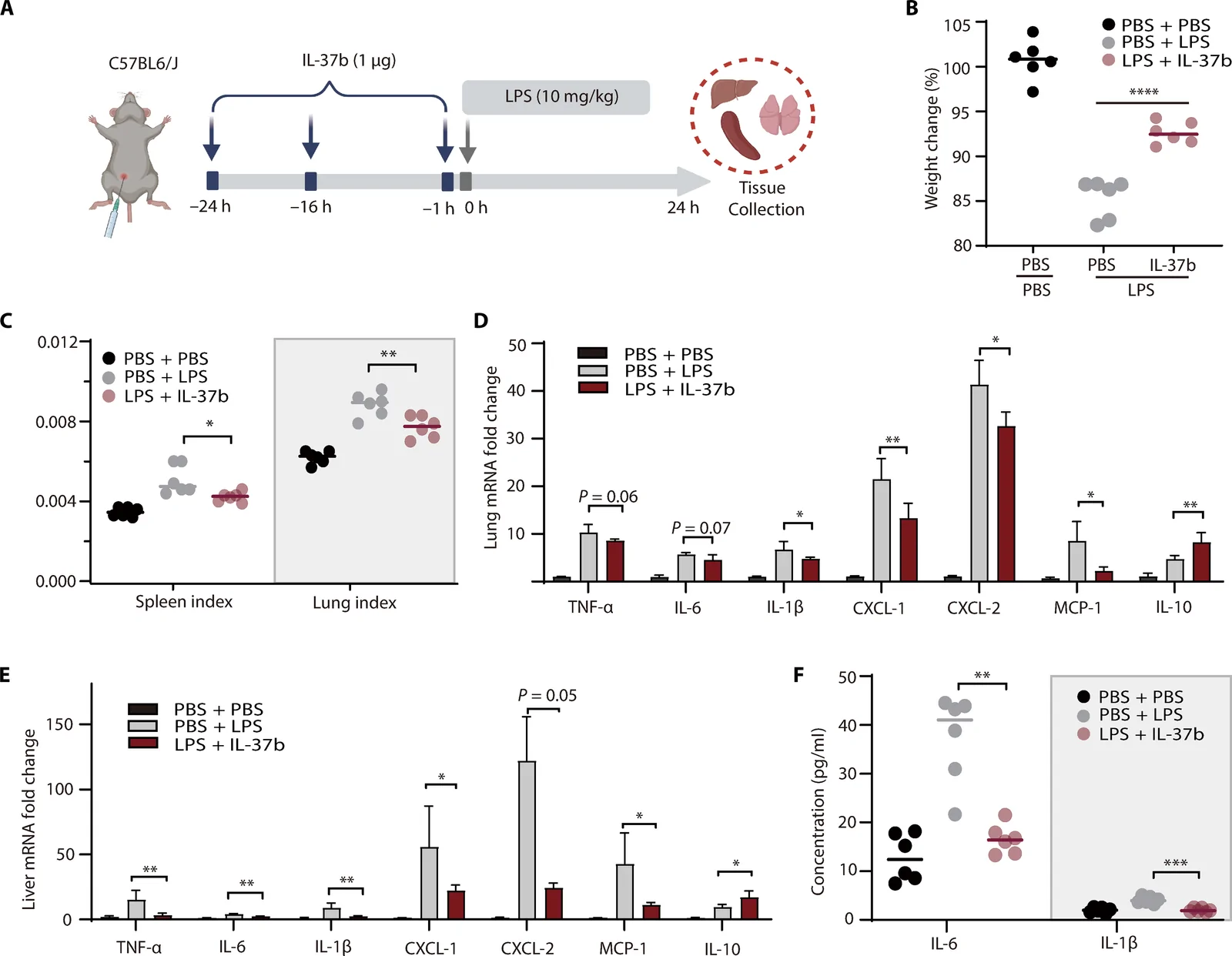
Low-dose anti-inflammatory activity of interleukin-37b (IL-37b). (A) Schematic of lipopolysaccharide (LPS)-induced sepsis model and IL-37b administration method (see text for details). (B) Percentage change in body weight of mice before and after administration of LPS intraperitoneally for 24 h (*n* = 6). (C) Spleen and lung indices for the indicated experimental groups (*n* = 6). (D) Relative expression of inflammation-related genes in mouse lung (*n* = 6). (E) Relative expression of inflammation-related genes in mouse liver (*n* = 6). (F) Serum levels of IL-6 and IL-1β measured by enzyme-linked immunosorbent assay (*n* = 6). *P < 0.05; **P < 0.01; ***P < 0.001; ****P < 0.0001.

Because cytokine-based immunomodulation in the gut is constrained by rapid degradation and limited local exposure, we first asked whether IL-37b retained measurable anti-inflammatory activity at very low doses and whether such activity could be observed in intestinal cells [19]. IL-37b, a recently identified anti-inflammatory cytokine lacking murine homologs, has demonstrated potent immunomodulatory activity at rather low doses (picogram range) in limited settings [16]. However, its anti-inflammatory efficacy has only been studied in limited models, with its actions in vivo and on intestinal cells remaining unclear. To address this gap, we expressed recombinant IL-37b and evaluated its anti-inflammatory activity using both in vivo and in vitro LPS-induced inflammation models. As schematically shown in Fig. 1A, an acute systemic inflammation model was first established by LPS via intraperitoneal injection to examine the efficacy of IL-37b. After the respective interventions, the purified recombinant IL-37b (Fig. S1A to C) alleviated body weight loss and organ pathological damage in mice (Fig. 1B and C). Consistent with these observations, IL-37b reduced or showed a suppressive trend in the expression of proinflammatory cytokines, including tumor necrosis factor-α (TNF-α), IL-6, IL-1β, CXCL-1, CXCL-2, and monocyte chemoattractant protein-1 (MCP-1), in the lung, liver, and spleen, while IL-10 expression (Fig. 1D and E and Fig. S1D) exhibited an upward trend. Consistently, IL-6 and IL-1β levels were also decreased in mouse serum compared to vehicle-treated controls (Fig. 1F). To gain an in-depth understanding of the anti-inflammatory activity of IL-37b, we further expanded the in vitro validation experiments using RAW264.7 macrophages and primary cells stimulated with LPS. Notably, IL-37b exhibited anti-inflammatory activity in immune cells (Fig. S1E and F) at low doses and also exerted anti-inflammatory effects on primary cells dissociated from digested mouse intestinal tissues (Fig. S1G to I) at picogram concentrations. Together, these results indicate that recombinant IL-37b is active in both in vivo and in vitro inflammatory models and retains anti-inflammatory activity in intestinal primary cells at picogram doses.

### Engineering a hypervesiculating EcN chassis for inducible IL-37b nanovesicle production

**Fig. 2.**
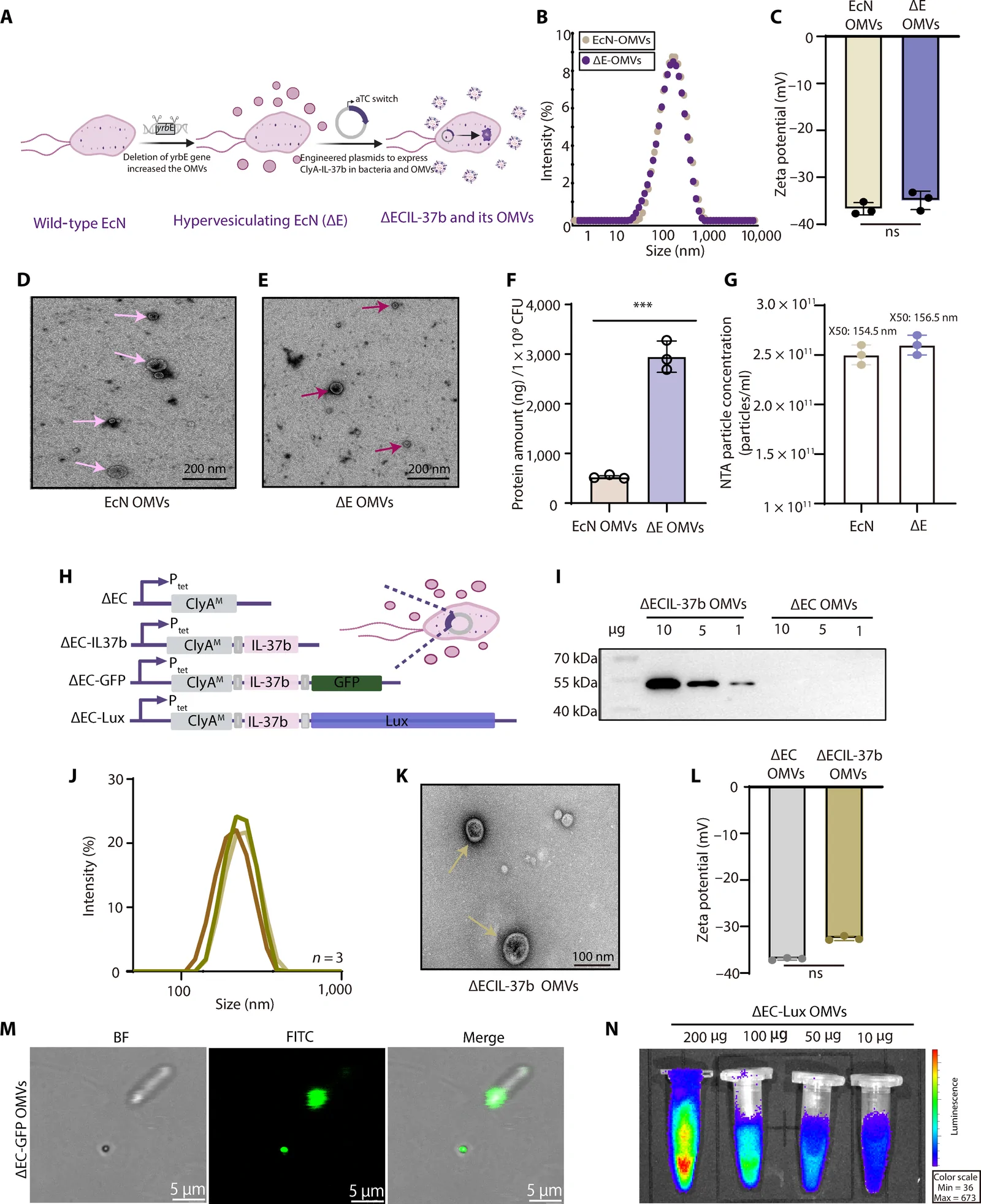
Construction and characterization of a hypervesiculating *Escherichia coli* Nissle 1917 (EcN) chassis for interleukin-37b (IL-37b)-bearing nanovesicle production. (A) Schematic diagram of IL-37b loading into a hypervesiculating EcN (ΔE) chassis. (B) Particle size averages of EcN outer membrane vesicles (OMVs) and ΔE OMVs. (C) Zeta potentials of EcN OMVs and ΔE OMVs. (D and E) Transmission electron microscopy (TEM) photomicrographs of EcN OMVs and ΔE OMVs. Scale bars, 200 nm. (F) OMVs from EcN and ΔE were quantified by Bicinchoninic acid (BCA) assay, with protein concentrations converted to total mass, normalized to input colony-forming units (CFU), and expressed as nanograms per 10^9^ CFU. (G) Nanoparticle tracking analysis (NTA) particle concentrations of vesicles derived from EcN and ΔE. (H) Schematic illustration of the engineered plasmid genetic construct. (I) Immunoblotting analysis of IL-37b localized on OMVs secreted by ΔECIL-37b (ΔE expressing the modified ClyA^M^-IL-37b fusion) and ΔEC (ΔE expressing modified ClyA^M^). (J) Particle size distribution profiles of ΔECIL-37b OMVs. (K) TEM photomicrograph of ΔECIL-37b OMVs. Scale bar, 100 nm. (L) Zeta potentials of ΔEC OMVs and ΔECIL-37b OMVs. (M) Confocal microscopy imaging of green fluorescent protein (GFP) displayed on OMVs secreted by ΔEC-GFP (ΔE expressing modified ClyA^M^-GFP). (N) Bioluminescence imaging of LuxCDABE signal anchored on OMVs secreted by ΔEC-Lux (ΔE expressing modified ClyA^M^-LuxCDABE). Data are presented as means ± SD. ***P < 0.001. ns, not significant; BF, bright field; FITC, fluorescein isothiocyanate.

Given the potent low-dose anti-inflammatory activity of IL-37b, our next goal is to maximize its translational potential for in vivo application. To this end, we designed its integration into a probiotic chassis, which may contribute to colitis mitigation by combining the merits of IL-37b with the probiotic properties of the chassis. Therefore, we optimized the EcN chassis to enable efficient production and display of IL-37b-bearing nanovesicles for local intestinal delivery (Fig. 2A). First, the probiotic EcN was genetically manipulated to remove the *yrbE* gene, a membrane-associated component of the VacJ/Yrb ATP-binding cassette transporter system, from the genome to yield a hypervesiculating strain ΔE (Fig. S2A and B). This manipulation was designed to enhance OMV production in the chassis strain (ΔE) [20]. Using the resulting engineered chassis strain, we observed that ΔE-derived OMVs retained nanoscale size distribution, negative surface charge, and typical vesicular morphology (Fig. 2B to E). Bicinchoninic acid (BCA)-based quantification showed increased OMV yield from ΔE compared with wild-type EcN (Fig. 2F). Nanoparticle tracking analysis (NTA) of BCA-normalized OMV preparations showed comparable particle concentrations between EcN OMVs and ΔE OMVs, suggesting no obvious particle-input bias after protein normalization (Fig. 2G).

We next engineered functional plasmids based on a modified cytolysin A (ClyA) anchoring protein, with cytolytic activity abolished by mutations at residues 182 to 184, under the control of a tetracycline (Tet) promoter [21]. In addition to IL-37b, plasmids encoding green fluorescent protein (GFP) and the bioluminescent enzyme complex LuxCDABE were also constructed to evaluate the versatility of the delivery platform and to enable cargo visualization and in vivo tracking (Fig. S2C to E). These plasmids were electroporated into the hypervesiculating ΔE strain to generate ΔEC (ΔE expressing modified ClyA^M^), ΔECIL-37b, ΔEC-GFP, and ΔEC-Lux (ΔE expressing modified ClyA^M^-LuxCDABE) (Fig. 2H). Characterization of the engineered strains and their vesicle-associated cargos showed that (a) plasmid introduction caused moderate differences in growth kinetics, while ΔE and ΔECIL-37b retained growth under the tested GI stress conditions without obvious anhydrotetracycline (aTC)-induced burden (Fig. S3); (b) IL-37b was displayed on the OMV surface, as evidenced by proteinase K sensitivity, and accounted for ~1% of total OMV protein mass, while the resulting ΔECIL-37b OMVs exhibited a slightly increased hydrodynamic diameter, negative surface charge, and typical vesicular morphology (Fig. 2I to L and Fig. S4A and B); and (c) ΔECIL-37b OMVs caused no obvious Caco-2 cytotoxicity or hemolysis at a wide panel of concentrations (Fig. S4C and D). Moreover, both GFP fluorescence (Fig. 2M) and Lux-derived bioluminescence (Fig. 2N) carried by OMVs were readily detected. Collectively, these data indicate that the engineered EcN chassis supports increased vesicle production and efficient surface presentation of IL-37b, while also accommodating additional protein cargos for vesicle-based display and delivery.

### Engineered-probiotic-derived nanovesicles enable in situ delivery of IL-37b in the intestine

**Fig. 3.**
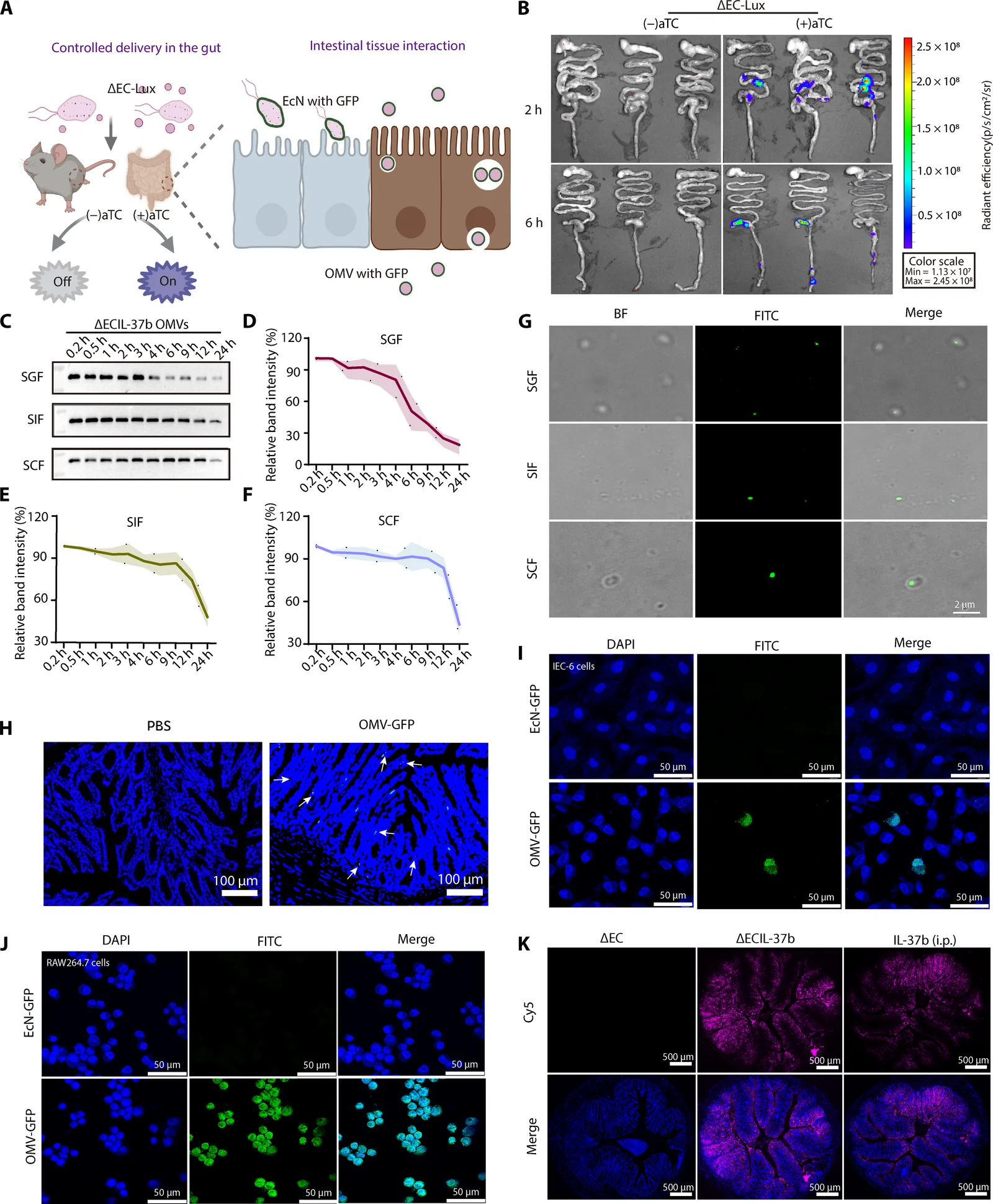
In vivo delivery characteristics of interleukin-37b (IL-37b) by engineered probiotics and their secreted outer membrane vesicles (OMVs). (A) Schematic diagram of the engineered probiotic delivery platform illustrating in vivo controllability, tissue penetration, and cargo in situ expression. (B) Ex vivo bioluminescence imaging (in vivo imaging system) of harvested gastrointestinal tissues from mice after oral gavage with ΔEC-Lux (ΔE expressing modified ClyA^M^–LuxCDABE) ± anhydrotetracycline (aTC) (*n* = 3). (C) Western blot analysis of OMV-associated IL-37b signals after exposure to simulated digestive fluids. (D to F) Quantification of IL-37b band intensity after exposure to simulated gastric, intestinal, and colonic fluids. (G) Confocal microscopy of green fluorescent protein (GFP)-labeled OMV signals after exposure to simulated gastric, intestinal, and colonic fluids. (H) Representative colon tissue sections from healthy mice after intracolonic administration of GFP-labeled OMVs or phosphate-buffered saline (PBS). Nuclei were counterstained with 4′,6-diamidino-2-phenylindole (DAPI). Scale bars, 100 μm. (I) Uptake of OMV-GFP and *Escherichia coli* Nissle 1917 (EcN)-GFP by IEC-6 cells. Scale bars, 50 μm. (J) Uptake of OMV-GFP and EcN-GFP by RAW264.7 cells. Scale bars, 50 μm. (K) Immunofluorescence detection of in situ delivered IL-37b signals from engineered bacteria in intestinal tissue. Scale bars, 500 μm. Data are presented as means ± SD. i.p., intraperitoneally.

Having confirmed IL-37b surface presentation on probiotic-derived nanovesicles in vitro, we next examined whether the ΔECIL-37b system could support local intestinal delivery in vivo (Fig. 3A). Ex vivo bioluminescence imaging of harvested GI tissues from mice treated with the ΔEC-Lux reporter strain carrying the same inducible expression architecture revealed intestinal luminescence after aTC induction, supporting inducible cargo expression in the gut (Fig. 3B). 1,1′-dioctadecyl-3,3,3′,3′-tetramethylindotricarbocyanine iodide (DiR)-based tracing was further used as a preliminary whole-organ fluorescence readout (Fig. S5), and we therefore performed subsequent microscopic assays to further evaluate vesicle-associated cargo retention, mucosal penetration, cellular uptake, and local IL-37b delivery. In simulated digestive fluids, GFP-labeled OMV signals and OMV-associated IL-37b signals remained detectable at early time points but decreased over time (Fig. 3C to G). These results suggest partial tolerance of vesicle-associated cargo to simulated GI conditions, with detectable signals persisting to some extent. Based on these observations, we further investigated the intestinal local penetration capability of OMVs. Intraluminal administration of GFP-labeled OMVs into the ligated colon revealed detectable GFP-positive signals within the colonic mucosa (Fig. 3H). Cellular uptake studies further demonstrated that OMV-GFP entered intestinal epithelial cells (IEC-6) and macrophages (RAW264.7) more efficiently than the parental EcN-GFP strain (Fig. 3I and J), highlighting their enhanced cellular delivery capacity. Finally, immunofluorescence analysis of colon tissues showed clear IL-37b-positive signals in mice treated with oral ΔECIL-37b, whereas the ΔEC empty vector control showed only weak background signal. Intraperitoneally administered IL-37b was included as a positive control (Fig. 3K). Overall, these findings support inducible intestinal expression and vesicle-associated local delivery of IL-37b by the ΔECIL-37b system.

### ΔECIL-37b ameliorates acute DSS colitis

**Fig. 4.**
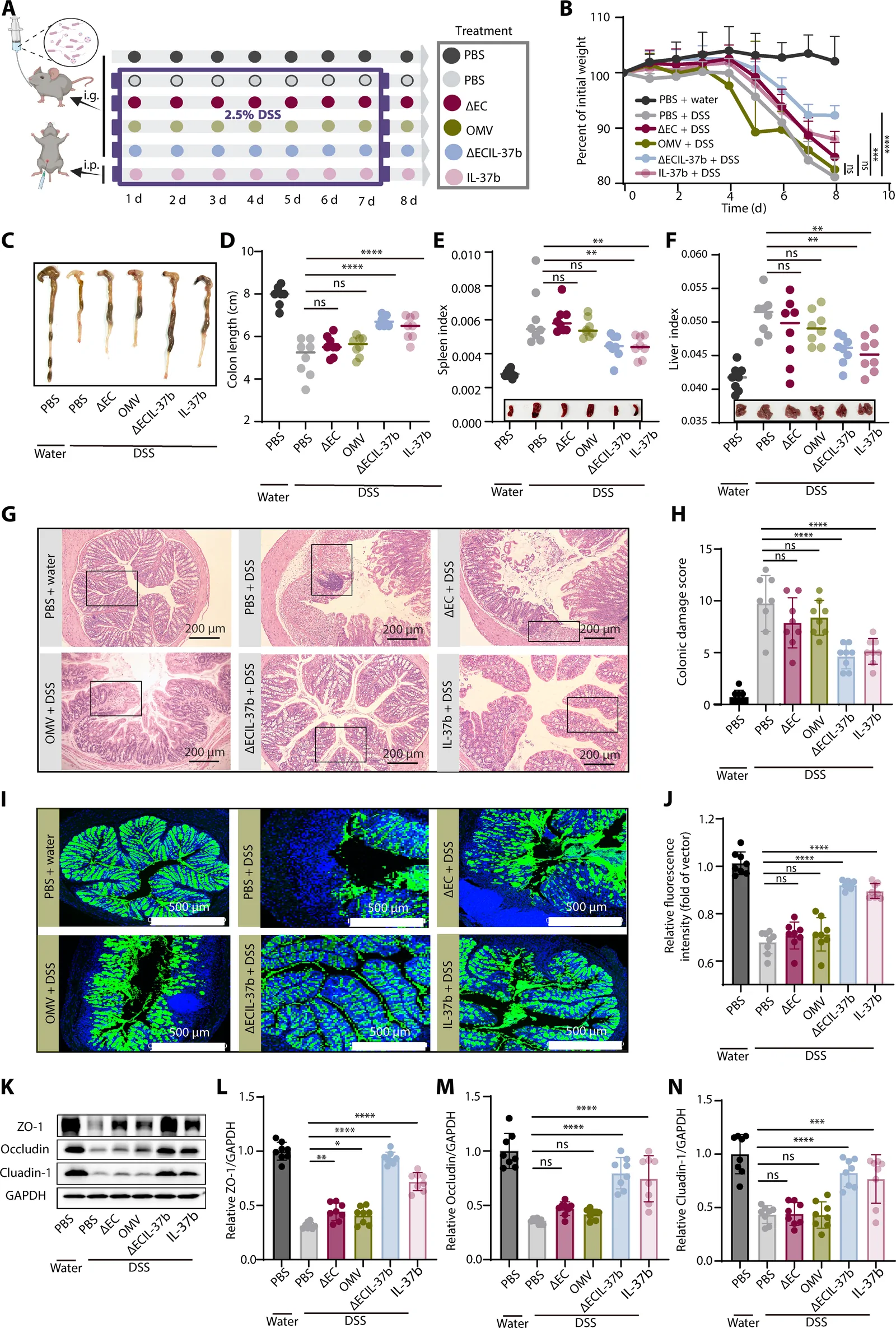
Therapeutic efficacy of ΔECIL-37b (ΔE expressing the modified ClyA^M^-IL-37b fusion) in acute dextran sodium sulfate (DSS) colitis. (A) Schematic of experimental design for DSS-induced colitis treatment. (B) Body weight dynamics across treatment groups (*n* = 8). (C) Representative colon photographs and (D) quantified colon lengths (*n* = 8). (E and F) Spleen and liver indices (organ weight/body weight ratio) (*n* = 8). (G) Representative hematoxylin and eosin (H&E)–stained colon sections from mice. Scale bars, 200 μm. Boxed areas indicate regions with prominent pathological lesions or treatment-associated changes. (H) Quantification of colonic damage using the histological grading scheme for DSS colitis (Table S6), in which inflammation (0 to 2), depth of injury (0 to 3), crypt damage (0 to 4), and percent involvement (1 to 4) were scored, and the total colonic damage score was calculated as the sum of these parameters. (I) Immunofluorescence analysis of mucin 2 (MUC2) in colon sections (nuclei counterstained with 4′,6-diamidino-2-phenylindole [DAPI]). Scale bars, 500 μm. (J) Quantification of MUC2 fluorescence intensity (*n* = 8). (K) Western blot analysis of tight junction proteins in colon tissues. (L to N) Quantification of zonula occludens-1 (ZO-1), Occludin, and Claudin-1 protein levels normalized to glyceraldehyde-3-phosphate dehydrogenase (GAPDH) (*n* = 8). *P < 0.05; **P < 0.01; ***P < 0.001; ****P < 0.0001. i.g., intragastrically.

Having established that ΔECIL-37b supports local intestinal delivery of IL-37b, we next evaluated its therapeutic efficacy in acute colitis (Fig. 4A). Acute colitis was induced by administration of 2.5% (w/v) dextran sodium sulfate (DSS) in drinking water for 7 d, during which mice received phosphate-buffered saline (PBS), the vector control (ΔEC), the OMV control, or ΔECIL-37b. Purified IL-37b administered by intraperitoneal injection was included as a positive control. After treatment, both ΔECIL-37b and purified IL-37b mitigated body weight loss compared with the other groups. The ΔEC and OMV control groups showed little to no significant effect (Fig. 4B). Consistent with this observation, mice receiving either ΔECIL-37b or IL-37b exhibited significantly less colon shortening (Fig. 4C and D). We next assessed systemic and histologic indices of disease severity. As expected, treatment with ΔECIL-37b or IL-37b significantly decreased spleen and liver indices compared with the DSS control (Fig. 4E and F). Histological assessment based on the DSS colitis grading scheme (Table S6) showed that DSS challenge induced prominent colonic injury, manifested by increased inflammatory cell infiltration, marked crypt damage/crypt loss, and extensive lesion involvement, with injury extending beyond the superficial mucosa in representative sections [22]. In contrast, treatment with ΔECIL-37b or purified IL-37b alleviated these pathological changes, preserving crypt architecture and limiting lesion extent, which was consistent with reduced total colonic damage scores. By comparison, ΔEC or OMV alone provided only modest to negligible improvement relative to the DSS control (Fig. 4G and H). In agreement with the histologic findings, the ΔECIL-37b and IL-37b groups significantly reduced the relative mRNA expression levels of the proinflammatory factors TNF-α, IL-6, and IL-1β, whereas the ΔEC group exhibited a moderate effect, and the OMV group consistently showed no reduction (Fig. S6A to C). Because acute DSS colitis is accompanied by barrier disruption, we next examined whether ΔECIL-37b could preserve colonic epithelial barrier integrity. Immunofluorescence staining showed that both ΔECIL-37b and IL-37b treatment increased mucin 2 (MUC2) expression compared with the colitis control (Fig. 4I and J). Compared with ΔEC or OMVs, ΔECIL-37b and IL-37b significantly up-regulated the expression of these tight junction proteins (Fig. 4K to N). Altogether, these findings indicate that ΔECIL-37b effectively attenuates DSS-induced acute colitis and promotes restoration of epithelial barrier integrity.

### ΔECIL-37b alleviates chronic colitis and protects gut microbial homeostasis

**Fig. 5.**
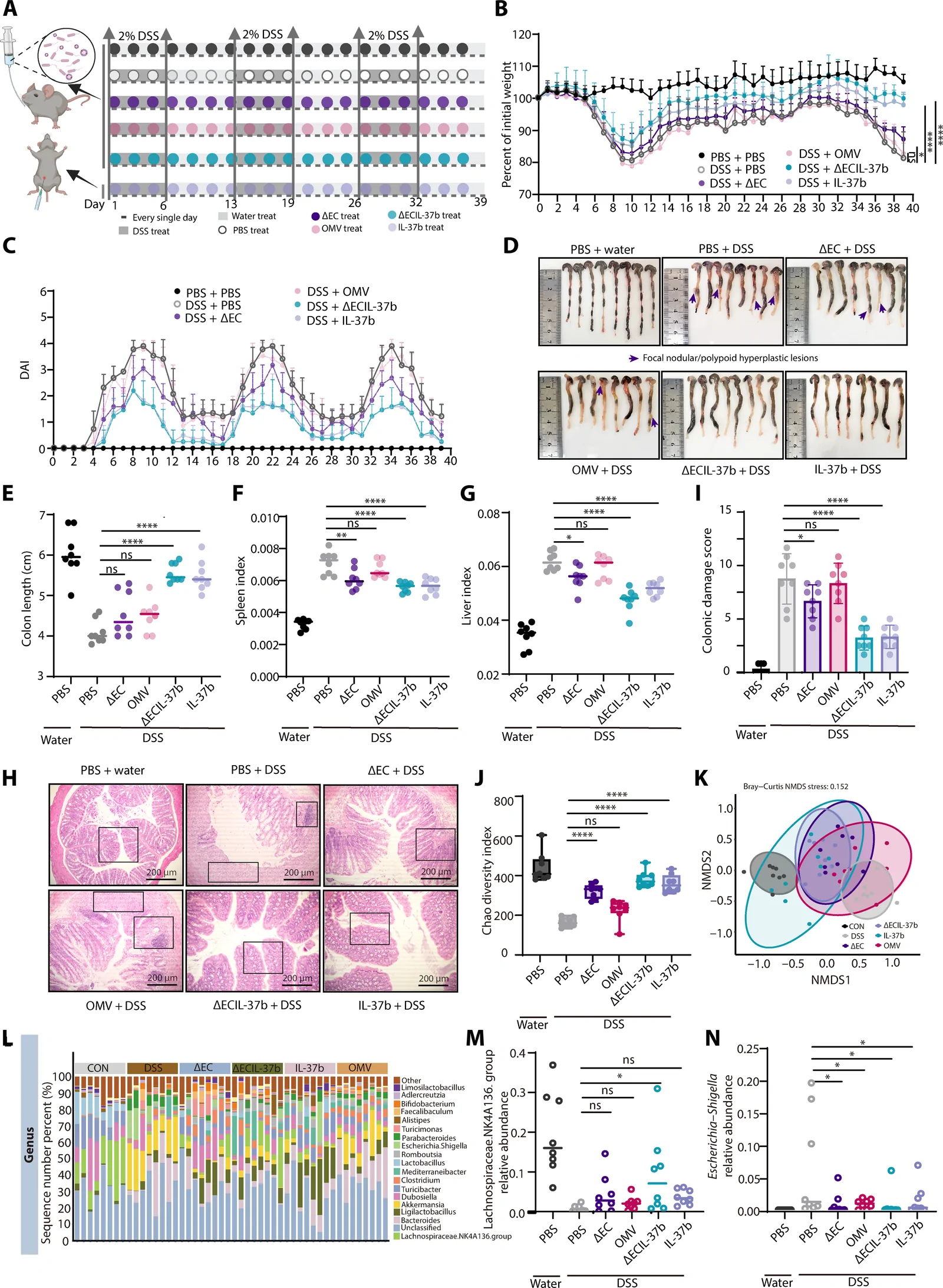
Therapeutic efficacy of ΔECIL-37b (ΔE expressing the modified ClyA^M^-IL-37b fusion) in chronic dextran sodium sulfate (DSS) colitis and associated gut microbiota remodeling. (A) Schematic of experimental design for chronic colitis treatment. (B) Body weight changes across treatment groups during the 39-d experimental period. (C) Changes in the disease activity index (DAI) among experimental groups. (D) Representative colon images and (E) quantified colon lengths. (F and G) Spleen and liver indices (organ weight/body weight ratio). (H) Representative hematoxylin and eosin (H&E)-stained colon sections from mice. Scale bars, 200 μm. Boxed areas indicate regions with prominent pathological lesions or treatment-associated changes. (I) Quantification of colonic damage using the histological grading scheme for DSS colitis (see Table S6 for criteria). (J) Chao index of the gut microbiota in different groups. (K) Nonmetric multidimensional scaling (NMDS) score plot based on Bray–Curtis. (L) Relative taxonomic composition of the gut microbiota at the genus level. Relative abundance of (M) Lachnospiraceae_NK4A136_group and (N) *Escherichia–Shigella*. Genus-level comparisons in (M) and (N) were adjusted using Benjamini–Hochberg false discovery rate (FDR) correction. For quantitative analyses, *n* = 8 biologically independent mice or fecal samples per group unless otherwise indicated. β-diversity differences in (K) were assessed by pairwise permutational multivariate analysis of variance (PERMANOVA) based on Bray–Curtis distance, with full results provided in Table S8. Data are presented as means ± SD. *P < 0.05; **P < 0.01; ****P < 0.0001.

To determine whether the therapeutic benefit of ΔECIL-37b could be sustained in a disease setting more closely resembling relapsing ulcerative colitis, we established a DSS-induced chronic colitis model using 3 cycles of DSS exposure and recovery (Fig. 5A). In this model, ΔECIL-37b attenuated DSS-induced body weight loss and reduced disease activity index (DAI) scores (detailed scoring criteria are shown in Table S7) [23], whereas ΔEC showed only limited protection and OMV treatment provided minimal protection (Fig. 5B and C). Consistent with these findings, ΔECIL-37b also alleviated gross colitis-associated abnormalities, including colon shortening and focal nodular/polypoid lesions, and reduced DSS-induced increases in spleen and liver indices (Fig. 5D to G). Histological analysis further showed that ΔECIL-37b attenuated chronic colonic injury, as reflected by improved preservation of crypt/glandular architecture and reduced inflammatory damage, consistent with lower colonic damage scores (Fig. 5H and I). Barrier-associated markers in the chronic model, including MUC2, zonula occludens-1 (ZO-1), Occludin, and Claudin-1, showed a similar restoration trend following ΔECIL-37b treatment (Fig. S7A to E). Because chronic colitis involves prolonged disruption of intestinal homeostasis and host–microbiota interactions [24], we next examined whether the therapeutic benefit of ΔECIL-37b was accompanied by preservation of the gut microbiota homeostasis. 16S ribosomal RNA (rRNA) gene sequencing analysis showed that ΔECIL-37b altered α-diversity, as indicated by the Chao index (Fig. 5J), and shifted the overall microbial community structure, as shown by β-diversity analysis (Fig. 5K). Pairwise permutational multivariate analysis of variance (PERMANOVA) based on Bray–Curtis distance further supported a significant community-level difference between the DSS and ΔECIL-37b groups (pseudo-*F* = 3.0229, *P* = 0.001, *q* = 0.00375; Table S8). At the genus level, ΔECIL-37b treatment altered the relative taxonomic composition of the gut microbiota (Fig. 5L) and was associated with increased Lachnospiraceae_NK4A136_group and decreased *Escherichia–Shigella* compared with the DSS group, and these differences remained significant after Benjamini–Hochberg false discovery rate (FDR) correction (Fig. 5M and N). Additional analyses of operational taxonomic unit (OTU) overlap, Shannon diversity, and phylum-level composition showed directionally consistent trends and are provided in Fig. S7F to J. These findings indicate that ΔECIL-37b not only alleviates chronic colitis but also fosters the microbial diversity and composition to a larger extent during colitis.

### ΔECIL-37b attenuates MyD88-related downstream ERK/NF-κB signaling and inflammatory-immune cross-talk

**Fig. 6.**
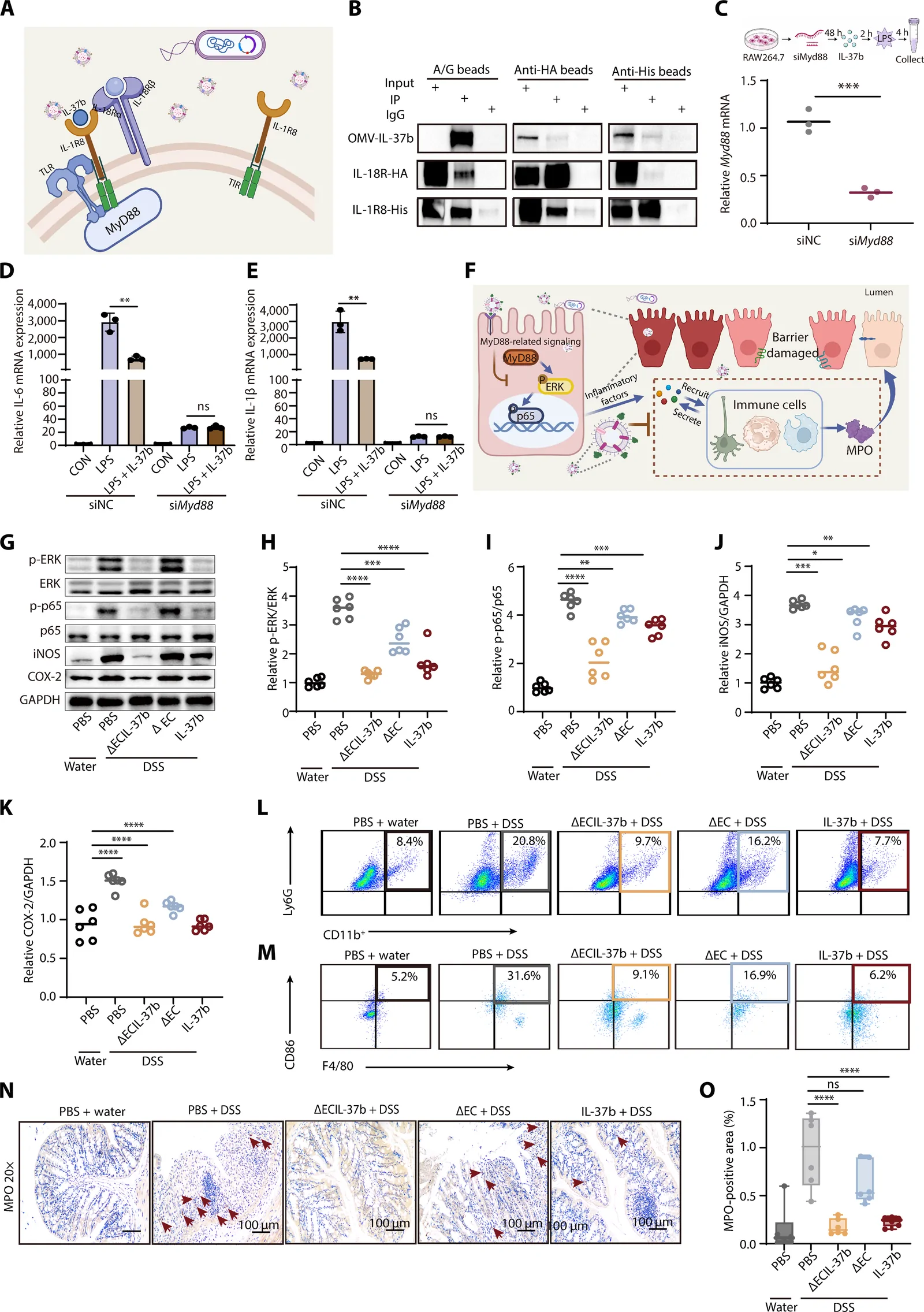
Attenuation of myeloid differentiation primary response 88 (MyD88)-related downstream extracellular-signal-regulated kinase (ERK)/nuclear factor κB (NF-κB) signaling and inflammatory-immune cross-talk by ΔECIL-37b (ΔE expressing the modified ClyA^M^-IL-37b fusion). (A) Schematic of interleukin-37b (IL-37b) receptor interaction mechanism. (B) Coimmunoprecipitation (Co-IP) and Western blot analysis of the interaction between engineered-bacteria-derived outer membrane vesicle (OMV)-IL-37b and IL-18 receptor α (IL-18Rα)/IL-1R8. (C) *Myd88* knockdown efficiency in RAW264.7 cells. (D and E) Relative IL-6 and IL-1β expression in control or *Myd88*-knockdown RAW264.7 cells after lipopolysaccharide (LPS) stimulation with or without IL-37b treatment. (F) Schematic of engineered bacteria attenuating inflammatory storm to restore intestinal barrier integrity. (G to K) Western blot analysis of colonic tissue protein levels for phosphorylation of ERK (p-ERK), ERK, p-p65, p65, inducible nitric oxide synthase (iNOS), cyclooxygenase-2 (COX-2), and glyceraldehyde-3-phosphate dehydrogenase (GAPDH) for the indicated experimental treatment groups. Quantification of p-ERK/ERK, p-p65/p65, iNOS/GAPDH, and COX-2/GAPDH is shown in (H) to (K). (L) Quantification of neutrophil infiltration in colonic lamina propria. (M) Quantification of macrophage infiltration in colonic lamina propria. (N) Representative immunohistochemistry (IHC) staining of myeloperoxidase (MPO) in colonic sections from the indicated groups. Arrowheads highlight representative punctate MPO-positive signals. Scale bars, 100 μm. (O) Corresponding quantitative analysis of MPO. *P < 0.05; **P < 0.01; ***P < 0.001; ****P < 0.0001. TLR, Toll-like receptor.

Given the therapeutic efficacy of ΔECIL-37b in colitis, we next investigated the signaling mechanisms underlying its anti-inflammatory and barrier-protective effects. Prior studies have demonstrated that IL-37 binds IL-18Rα and recruits IL-1R8 to form the ternary IL-37–IL-18Rα–IL-1R8 complex on the cell surface. IL-1R8 contains a Toll/IL-1 receptor (TIR) domain, and ligand binding is thought to alter TIR-domain-associated signaling [25]. This interaction is thought to attenuate TIR-domain-associated myeloid differentiation primary response 88 (MyD88)-related signaling (Fig. 6A) [26]. We next asked whether OMV-associated IL-37b could interact with the reported receptor components and whether MyD88-related signaling was involved in the anti-inflammatory activity of IL-37b. As a supporting control, recombinant IL-37b showed detectable association with IL-18Rα and IL-1R8 in the coimmunoprecipitation (Co-IP) assay (Fig. S8D). We further performed Co-IP using engineered-bacteria-derived OMV-IL-37b, which also showed association with IL-18Rα and IL-1R8 (Fig. 6B). To further examine the involvement of MyD88-related signaling, we performed *Myd88* knockdown in RAW264.7 cells. *Myd88* knockdown reduced LPS-induced inflammatory gene expression and largely diminished the additional inhibitory effect of IL-37b on IL-6 and IL-1β expression (Fig. 6C to E). Supplementary Western blot and immunohistochemistry (IHC) quantification showed reduced DSS-associated MyD88 protein signals after ΔECIL-37b or IL-37b treatment (Fig. S9). We next examined extracellular-signal-regulated kinase (ERK)/nuclear factor κB (NF-κB) signaling downstream of MyD88-related inflammatory activation (Fig. 6F) [27]. As shown in Fig. 6G to K, compared with the colitis mice, both ΔECIL-37b and IL-37b significantly suppressed the phosphorylation of ERK (p-ERK) and p65, accompanied by reduced expression of inducible nitric oxide synthase (iNOS) and cyclooxygenase-2 (COX-2) and decreased relative mRNA expression levels of the proinflammatory cytokines TNF-α (Fig. S8E), IL-6 (Fig. S8F), and IL-1β (Fig. S8G). Although the ΔEC group also exhibited a moderate effect relative to the colitis control, its effect was much weaker than that in mice administered ΔECIL-37b. Consistently, pharmacological validation using the MyD88 dimerization inhibitor ST2825 and the activator phorbol 12-myristate 13-acetate further supported the involvement of this pathway (Fig. S10). We next examined immune cell infiltration and associated myeloperoxidase (MPO) expression in the inflamed intestine, as infiltrating inflammatory myeloid cells can exacerbate epithelial injury through MPO-associated effector activity and may therefore link immune cell recruitment to barrier disruption [28]. Based on the gating strategy shown in Fig. S11A, flow cytometry indicated that both ΔECIL-37b and purified IL-37b suppressed the infiltration of neutrophils (Fig. 6L), macrophages (Fig. 6M), and dendritic cells (Fig. S11B) in intestinal tissues. We then assessed MPO expression in colonic tissues by IHC analysis and found that ΔECIL-37b markedly reduced MPO signals (Fig. 6N and O), consistent with reduced inflammatory tissue damage. These findings support that ΔECIL-37b attenuates MyD88-related downstream ERK/NF-κB signaling, thereby limiting inflammatory mediator release, modulating inflammatory-immune cross-talk, and reducing immune-cell-associated mucosal injury, ultimately promoting epithelial barrier restoration and intestinal homeostasis in colitis.

### ΔECIL-37b shows a favorable safety profile in healthy mice

To comprehensively profile the safety of the systems constructed in this study, healthy mice received PBS, EcN, ΔEC, or ΔECIL-37b daily for 7 d, followed by hematologic, biochemical, and histologic analyses (Fig. S12A). Compared with the PBS group, treatment with EcN, ΔEC, or ΔECIL-37b did not significantly alter hematologic indices, including white blood cell, red blood cell, platelet, and hemoglobin levels (Fig. S12B to I). Serum biochemical parameters associated with liver and kidney function, including alanine aminotransferase, aspartate aminotransferase, blood urea nitrogen, and creatinine, also remained unchanged across groups (Fig. S12J to M). Consistently, hematoxylin and eosin (H&E) staining of the heart, liver, spleen, lung, and kidney showed no obvious histopathologic abnormalities in any treatment group (Fig. S12N). To elucidate the long-term safety, we performed another 28-d repeated-dosing animal trial, and the results showed no obvious changes in body weight, hematological or biochemical parameters, major-organ histology (Fig. S13). In addition, serum anti-IL-37b antibody levels were examined, and no detectable antibody production was observed after long-term challenge (Fig. S14A). The colonization trial demonstrated that no prolonged uncontrolled intestinal persistence was observed (Fig. S14B). Throughout the animal trial, no obvious plasmid loss (Fig. S14C) or detectable horizontal transfer was observed (Fig. S14D). These findings support the favorable preliminary in vivo safety profile of ΔECIL-37b.

## Discussion

IL-37b is an anti-inflammatory member of the IL-1 cytokine family, and its increased epithelial expression in inflamed IBD mucosa supports its relevance to intestinal inflammation [29]. Although it is a relatively newly identified cytokine, IL-37b has garnered considerable attention due to its ability to exert anti-inflammatory effects on PBMCs at picogram concentrations [16]. Although no murine ortholog of IL-37 exists, prior studies suggest cross-species pharmacologic responsiveness, as transgenic mice expressing IL-37b are protected against colitis [30]. Importantly, our study focuses on the therapeutic activity of exogenously delivered human IL-37b, rather than the physiological role of endogenous IL-37 in mice. IL-37 has been reported to signal through IL-18Rα in cooperation with IL-1R8, and these receptor components are present and conserved in mice, enabling murine cells to respond to IL-37b. In the present study, IL-37b alleviated inflammation in both in vitro and in vivo settings, including protection in an LPS-induced endotoxemia model and anti-inflammatory effects in primary cells dissociated from mouse intestinal tissues, supporting the use of wild-type mice for pharmacologic evaluation of IL-37b delivery. Notably, the 2 cell models used required different therapeutic concentrations of IL-37b. This cell-type-dependent variation in efficacy may reflect differences in receptor abundance and distribution among cell types. Previous work has shown that IL-1R8, the receptor recruited by IL-37 to mediate its function, is more abundant in murine primary intestinal cells than in RAW264.7 cells. The amino acid sequence of IL-1R8 is also highly conserved between humans and mice, with 82% identity [31]. Together, these observations support the intestine as a favorable target tissue in which exogenous IL-37b can elicit anti-inflammatory responses even at relatively low doses.

The efficiency of oral drug delivery is, however, substantially constrained by the physiological barriers of the GI tract, including the hostile acidic environment and proteases in the stomach, as well as the intestinal mucus layer and epithelial barrier. Among the array of precision medicine approaches, emerging strategies such as antibody therapy [32], cell therapy [33], metabolic reprogramming [34], and tolerogenic vaccines [35] aim to overcome challenges including low drug bioavailability, insufficient targeting specificity, and transient therapeutic efficacy [36]. Nevertheless, these methods still require further refinement due to their complex manufacturing processes, unstable drug formulations, and high production costs. In this context, recent IBD studies have increasingly focused on locally acting platforms, including EcN/probiotic-derived engineered OMVs [37] and nanoformulations that release H_2_S [38] or CO [39], scavenge reactive oxygen species, reshape mucosal immunity, restore epithelial barrier integrity, or regulate gut microbiota. These advances highlight the therapeutic value of local microenvironment remodeling in IBD and support our rationale for developing a live engineered probiotic system that enables inducible intestinal production and vesicle-associated delivery of IL-37b. As a well-established probiotic, the EcN chassis exhibits adaptation to the GI environment, enabling it to survive GI transit and transiently persist in the intestine. Owing to this property, the IL-37b cargo is expected to be produced continuously in the gut and may prolong therapeutic efficacy compared with a single dose of purified IL-37b. Besides, this probiotic background may partially account for the protection observed in the ΔEC control group, while IL-37b loading adds an additional immunomodulatory component. This observation supports our design, which combines the advantages of the probiotic chassis and its IL-37b cargo. OMVs secreted in situ provide a nanoscale carrier that extends cargo delivery beyond bacterial colonization sites and supports penetration into intestinal mucosal tissues [40]. As a live-bacteria-based system, it is important to ensure biosafety and ecological safety throughout therapy. To this end, we genetically manipulated the chassis strain and monitored its biocompatibility over an extended period. As shown in the results above, no adverse effects or horizontal gene transfer were observed during ΔECIL-37b treatment, supporting its favorable preliminary in vivo safety profile, fully demonstrating its potential for in vivo application. Collectively, this strategy of directly engineering parent bacteria to generate modified OMVs integrates cargo production, vesicle biogenesis, and intestinal delivery within a single living system. In this way, the platform combines a gut-adapted bacterial chassis, local production of IL-37b-bearing vesicles, and the potent low-dose activity of IL-37b.

**Fig. 7.**
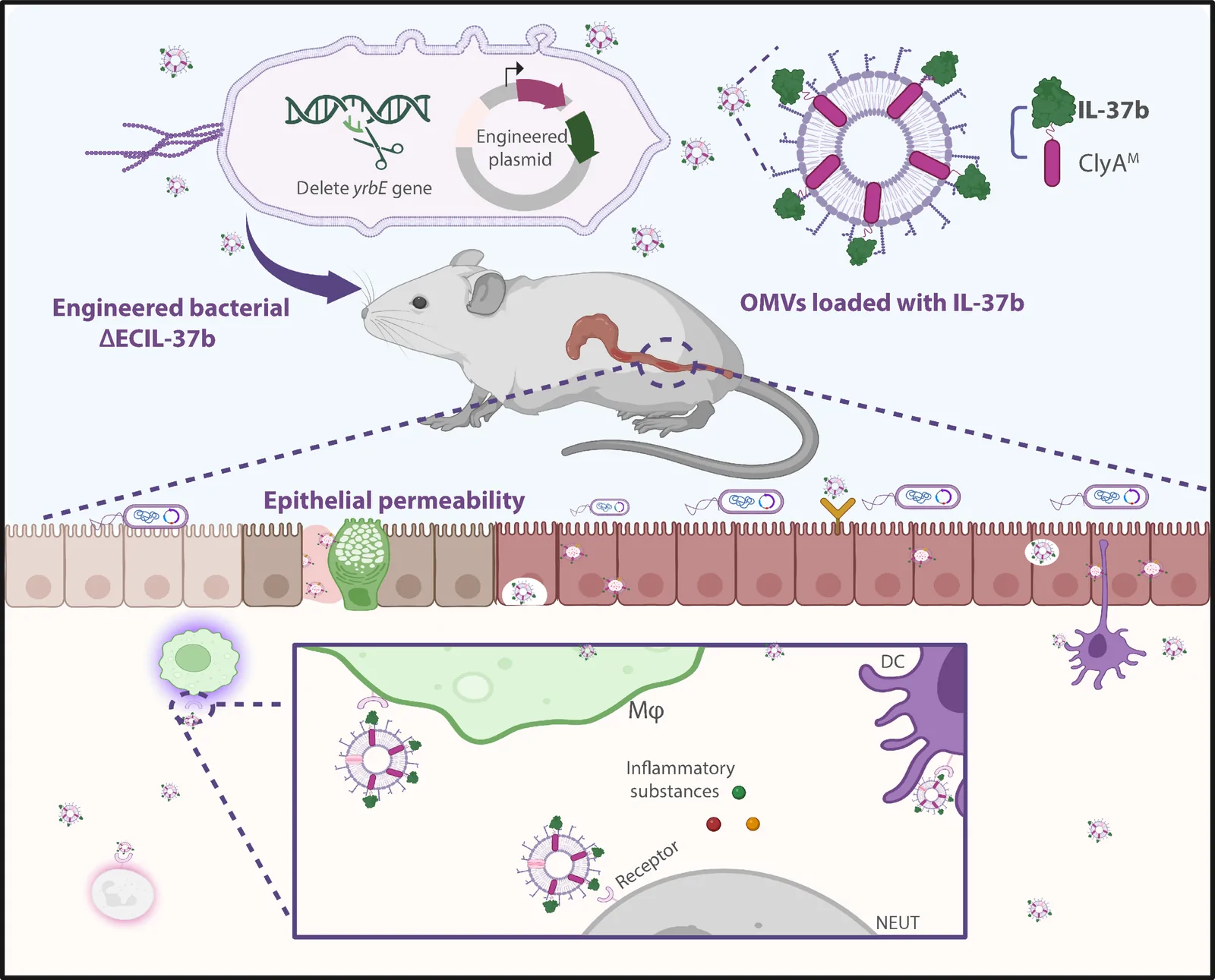
Schematic summary of the proposed mechanism underlying engineered-probiotic-mediated local IL-37b delivery and immunomodulation in colitis.

In this study, an acute colitis model was first established to simulate inflammatory damage to the intestinal epithelium and mucus layer. Under these conditions, the engineered strain ΔECIL-37b effectively alleviated pathological injury and barrier disruption and showed efficacy comparable to intraperitoneally administered IL-37b. In addition to this desirable efficacy, ΔECIL-37b supports the concept and feasibility of using an orally administered living probiotic as an alternative IL-37b delivery strategy. Compared with administration of purified recombinant IL-37b protein, ΔECIL-37b has several practical advantages. First, it can produce IL-37b in situ, avoiding the costly and technically fastidious procedures required for recombinant protein expression, purification, storage, and dosing. Second, ΔECIL-37b is administered orally, which is less invasive than intraperitoneal injection and may reduce treatment-associated stress to the host. Third, recombinant cytokine proteins are generally susceptible to degradation and rapid clearance in vivo, whereas an engineered probiotic has the potential to provide sustained local production of IL-37b in the intestinal environment during colonization. To better recapitulate the chronic and relapsing nature of clinical colitis and to evaluate the sustained therapeutic performance of IL-37b delivery throughout repeated injury–recovery cycles, we further assessed the efficacy of our engineered strain in a DSS-induced chronic colitis model. The results demonstrated that over 3 cycles of colitis induction and recovery, both the engineered probiotic ΔECIL-37b and purified IL-37b consistently protected the intestine from damage. In addition, ΔECIL-37b treatment was associated with partial restoration of microbial diversity, increased relative abundance of Lachnospiraceae_NK4A136_group, and decreased relative abundance of *Escherichia–Shigella*, suggesting remodeling of the gut microbiota during chronic disease. These findings were consistent with attenuation of MyD88-related downstream ERK/NF-κB signaling and reduced inflammatory-immune cross-talk. Although IL-37b is primarily considered to attenuate MyD88-related downstream signaling, reduced MyD88 protein expression has also been reported under inflammatory conditions, consistent with our findings [41,42]. Whether this reduction reflects direct regulation or a secondary consequence of inflammation attenuation remains unclear, and we therefore do not consider it the primary mechanism of IL-37b action. However, recent reports have shown that IL-37 transgenic mice may display increased susceptibility to colitis-associated colorectal cancer [43] or aggravated colitis linked to gut microbiota dysbiosis [44]. These contrasting findings suggest that the effects of IL-37b may be context dependent. In transgenic settings characterized by systemic, persistent, and unregulated overexpression, adverse outcomes may arise from the inability to precisely control the timing and localization of IL-37 activity. This concern highlights the importance of delivery strategies with improved spatial and temporal control. Although we initially constructed a constitutive IL-37b expression plasmid as a comparison module, we subsequently introduced an aTC-inducible module into ΔECIL-37b to reduce the risk of sustained protein expression and to lessen the fitness burden on the bacterial chassis. We therefore propose that spatial localization, temporal controllability, and delivery precision are important determinants of the intestinal protective effects observed with our engineered probiotic (Fig. 7).

However, OMVs alone did not confer significant intestinal protection in either acute or chronic colitis model. This biodistribution pattern, together with the use of a live engineered bacterial chassis, highlighted the need for systematic safety evaluation. We therefore performed short-term and repeated-dose in vivo safety assessments of the engineered probiotic. The results indicated that mice treated with the engineered strain showed no notable differences in complete blood count, serum biochemical parameters, or liver and kidney function indicators compared to PBS-treated controls, suggesting a favorable preliminary in vivo safety profile. Consistently, the 28-d repeated-dosing assay revealed no obvious hematological, biochemical, or major-organ histological abnormalities, no detectable serum anti-IL-37b antibody signals, no prolonged intestinal persistence, no obvious plasmid loss during the early observation period, and no detectable horizontal transfer under the tested conditions. Nevertheless, broader antidrug antibody profiling, extended safety evaluation, and neutralizing-antibody or IL-37b loss-of-function studies remain necessary to more directly define the cargo-specific contribution of IL-37b and the long-term safety profile of this system. In addition to these safety-related considerations, further mechanistic studies are warranted to determine whether OMV-delivered IL-37b engages additional signaling pathways and to dissect the relative contributions of live bacterial colonization, probiotic–host interactions, and OMV-mediated IL-37b delivery to the observed in vivo efficacy. Although IL-37b is not yet an established clinical therapeutic target for IBD, the present study was designed as a proof-of-concept evaluation of localized IL-37b delivery rather than as a direct substitute for approved biologics. Further studies are needed to define its therapeutic window, long-term safety, and translational relevance. In summary, this study presents an inducible engineered probiotic system for OMV-mediated IL-37b delivery, supporting its potential as a localized immunomodulatory strategy for intestinal inflammatory disease.

## Materials and Methods

### Study design

This study aimed to evaluate the anti-inflammatory efficacy, delivery characteristics, and biosafety of the engineered probiotic ΔECIL-37b in vitro and in vivo. Sample sizes for animal experiments were determined on the basis of our prior experience with DSS-induced colitis and related probiotic intervention studies, and group sizes are indicated in the corresponding sections and figure legends. Mice were randomly assigned to experimental groups. Sample collection, histological processing, and outcome assessment were performed by investigators blinded to group allocation unless otherwise specified. Data were unblinded at the stage of statistical analysis. No animals or samples were excluded from the analyses unless a predefined technical failure occurred. In vitro experiments were performed with at least 3 independent biological replicates unless otherwise stated, and the exact statistical tests used for each dataset are indicated in the figure legends.

### Reagents, cells, and animals

All chemicals and reagents were purchased from commercial sources and stored according to instructions. This study used the antibodies, strains, and plasmids listed in Tables S1 and S2. Primary intestinal cells were isolated from mouse intestine by collagenase digestion. Briefly, washed and cut mouse intestinal tissues were incubated in collagenase I (Solarbio, Beijing, China) solution (1 mg/ml for 1 h at 37 °C). The processed tissues were then washed and digested at least twice and passed through cell strainers as previously described [45]. The resulting cell preparation was used as mixed primary intestinal cells, and experiments were performed with 3 independent biological replicates after viability assessment. Murine macrophage RAW264.7 cells, intestinal epithelial cell line IEC-6, human intestinal epithelial Caco-2 cells, and human embryonic kidney (HEK) 293T cells were obtained from the cell bank of the type culture collection of the Chinese Academy of Sciences (Shanghai, China). Cells were incubated at 37 °C in 5% CO_2_ in Dulbecco’s modified Eagle’s medium supplemented with 10% fetal bovine serum and 1% penicillin–streptomycin.

Female C57BL/6J mice aged 7 to 8 weeks were obtained from BesTest Bio-Tech (Zhuhai, China). Mice were housed under specific-pathogen-free conditions, and sterile food and water were provided ad libitum.

### Preparation of engineered plasmids and engineered bacteria

Plasmids HM617-IL-37b (46 to 218)-Flag, HM617-IL-18R-hemagglutinin (HA), and HM617-IL-1R8-histidine (His) (see Fig. S8A to C) were synthesized by Tsingke Biotechnology Co. Ltd., for the purpose of transfection into HEK293T cells and subsequent validation by Co-IP. We used a CRISPR–Cas9 approach to delete the *yrbE* gene of EcN, and this strain was denoted ΔE (see Table S3). The engineered plasmid pTet-ClyA^M^-IL-37b was constructed using standard restriction enzyme and homologous recombination methods. Plasmid construction consisted of amplifying the *ClyA* sequence from *E. coli* strain MG1655, altering amino acids 182 to 184, and fusing the modified *ClyA^M^* sequence to the IL-37b gene with a linker. Subsequently, the promoter of the pET28a plasmid backbone was replaced with Tet repressor/operator (tetR/O), and the construct pTet-ClyA^M^-IL-37b was electroporated into ΔE to complete the construction of the engineered bacterium ΔECIL-37b. In addition, we constructed the engineering plasmids pTet-ClyA^M^, pTet-ClyA^M^-IL-37b-LuxCDABE, and pTet-ClyA^M^-GFP using the same method as described above and electroporated into ΔE strain to complete the preparation of ΔEC, ΔEC-Lux, and ΔEC-GFP (see Tables S2 and S4). Engineered strains were cultured in LB medium supplemented with the indicated concentrations of sodium deoxycholate or adjusted to different pH values, with or without aTC induction, and growth was monitored by optical density at 600 nm over 24 h.

### Heterologous expression and in vitro activity verification of IL-37b

The recombinant IL-37b (46 to 218) sequence (Miaoling Biology, Wuhan, China) was cloned into the expression vector pET28a. Recombinant IL-37b was purified by Ni–nitrilotriacetic acid affinity chromatography (GE Healthcare, Chicago, IL, USA), followed by gradient dialysis. The purified protein was processed using endotoxin removal and detection kits (GenScript Biotechnology Corporation, Nanjing, China) until the endotoxin level was <0.1 EU/μg for subsequent cell and animal experiments. Primary intestinal cells were seeded at a density of 1 × 10^6^ per well in 12-well plates containing culture medium. The cells were treated with recombinant IL-37b for 2 h at varying doses prior to stimulation with LPS (1 μg/ml; Sigma-Aldrich, St. Louis, MO, USA) for 12 h. The supernatant was then discarded, and the cells were rinsed with ice-cold PBS and lysed using 500 μl of cell lysis buffer (Vazyme, Nanjing, China); the extracts were stored at −20 °C. RAW264.7 cells were cultured under similar conditions and assays were carried out at 70% to 80% confluence. Prior to stimulation with LPS (1 μg/ml) for 4 h, each well was preincubated for 2 h with or without IL-37b at different concentrations. Cells were collected for total RNA extraction. To further pharmacologically validate the downstream signaling mechanism of IL-37b, RAW264.7 cells were pretreated with recombinant human IL-37b (100 ng/ml) for 4 h, followed by phorbol 12-myristate 13-acetate (25 nM; Sigma-Aldrich, St. Louis, MO, USA) for 30 min or ST2825 (5 μM; MedChemExpress, Monmouth Junction, NJ, USA) for 1 h before LPS stimulation. For quantitative real-time polymerase chain reaction (qRT-PCR), cells were harvested 4 h after LPS stimulation, and the expression of IL-6 and IL-1β was measured. For Western blotting, cells were harvested 15 min after LPS stimulation and analyzed for p-ERK, ERK, p-p65, and p65. Vehicle-treated cells were included where appropriate. For *Myd88* knockdown, RAW264.7 cells were transfected with *Myd88*-specific small interfering RNA (si*Myd88)* or control small interfering RNA for 48 h, pretreated with IL-37b for 2 h, and stimulated with LPS for 4 h before qRT-PCR analysis of *Myd88*, IL-6, and IL-1β expression.

### Isolation and characterization of OMVs

OMVs were extracted and isolated using sequential differential centrifugation as previously described [46]. Briefly, engineered bacteria were grown to late log phase in LB broth supplemented with aTC (200 ng/ml) and kanamycin (50 μg/ml). Following removal of bacteria by centrifugation, the supernatants were filtered, ultrafiltered, and ultracentrifuged, and the resulting OMV pellets were resuspended in cold PBS. The mean OMV particle size distribution and zeta potential were determined using a Zetasizer Nano ZS instrument (Malvern, Worcestershire, UK). The morphology of OMVs was imaged using a field emission transmission electron microscope (FEI Company, UK). For proteinase K protection assays, ΔECIL-37b OMVs were incubated with proteinase K in the presence or absence of EDTA under the indicated conditions, followed by Western blotting to assess the accessibility of OMV-associated IL-37b. BCA-normalized EcN OMV and ΔE OMV preparations were further analyzed by nanoparticle tracking analysis to determine particle concentration. To estimate IL-37b loading efficiency, defined amounts of ΔECIL-37b OMV proteins and recombinant IL-37b standards were analyzed by Western blotting, and band intensities were quantified by densitometry to calculate IL-37b content relative to total OMV protein input. OMV samples engineered to express IL-37b, GFP, and LuxCDABE were assessed by Western blotting, confocal microscopy using a Leica laser scanning confocal microscope (Wetzlar, Germany), and an in vivo imaging system. Caco-2 cells or mouse erythrocytes were incubated with ΔECIL-37b OMVs at the indicated concentrations. Cell viability was measured using a Cell Counting Kit-8 assay, and hemolysis was quantified by measuring supernatant absorbance, with PBS and Triton X-100 used as negative and positive controls, respectively.

### Controllable protein expression in engineered bacteria

To assess whether the recombinant IL-37b protein was expressed in vivo under aTC induction, we constructed LuxCDABE strains as described previously [47]. The mice were gavaged with 1 × 10^9^ colony-forming units (CFU) of ΔEC-Lux bacteria for 2 h prior to induction with or without aTC (0.1 mg/ml) for 2 and 6 h. The mice were then euthanized, and intestinal segments were dissected and imaged using a commercial in vivo imaging system.

### Analysis of in vivo properties of OMVs and their cargo proteins

To evaluate DiR-associated fluorescent tracing, DiR-labeled OMV-IL-37b or free DiR/PBS was administered orally to C57BL/6J mice, and harvested tissues were imaged using an in vivo imaging system. For region of interest (ROI) quantification, the GI tract, liver, spleen, and kidney were identified on the basis of anatomical location in the ex vivo images and analyzed using the same background correction and quantification workflow. After pixel-level background correction, integrated fluorescence intensity was calculated as the background-corrected mean ROI intensity multiplied by the ROI area. The free DiR in PBS control was included as a dye-associated background control, and the corresponding fluorescence intensity was subtracted from DiR-labeled OMV-IL-37b signals at each time point for background correction. Simulated gastric fluid (SGF), simulated intestinal fluid (SIF), and simulated colonic fluid (SCF) were prepared as per the Chinese Pharmacopoeia [48]. OMV-GFP and OMV-IL-37b were incubated in these fluids at 37 °C for the indicated times. The samples were then analyzed by confocal microscopy or Western blot.

Intestinal permeability of OMVs was evaluated using a colon ligation model. GFP-labeled OMVs were injected into the ligated colon lumen, and after 6 h, the colon tissues were harvested for cryosectioning and imaging by confocal microscopy. Cellular uptake of the OMVs was further observed. IEC-6 cells and RAW264.7 cells were cultured in 12-well plates for 24 h at 1 × 10^5^ per well. OMV uptake was assessed by incubating cells with EcN-GFP or OMV-GFP for 4 h. The cells were then rinsed with cold PBS and fixed in 4% paraformaldehyde solution and imaged.

### In situ delivery of IL-37b in the gut by engineered bacteria

C57BL/6J mice were divided into 3 groups, and after 1 week of the respective treatments (daily oral gavage of ΔEC, daily oral gavage of 1 × 10^9^ CFU of ΔECIL-37b, or daily intraperitoneal injection of 1 μg of IL-37b), mouse colons were harvested for cryosectioning and immunofluorescence analysis to visualize IL-37b-specific signals.

### Animal model

C57BL/6J mice were acclimatized to the rearing environment for 7 d and were randomly divided into 3 groups of 6 mice each. To verify the activity of IL-37b expressed in *E. coli*, mice were injected intraperitoneally with 1 μg of IL-37b or an equal volume of PBS. After 24 h, LPS (10 mg/kg) was injected intraperitoneally and 24 h later, the mice were euthanized, and serum and individual organs were collected for subsequent analyses.

We also used a DSS model of induced colitis using 48 female C57BL/6J mice aged 7 to 8 weeks, which were assigned to 6 groups (control, DSS + PBS, DSS + ΔEC, DSS + OMV, DSS + ΔECIL-37b, and DSS + IL-37b) with 8 mice each. The experimental period was 8 d. Mice received engineered bacteria or an equal volume of PBS by gavage, or 1 μg of IL-37b by intraperitoneal injection, daily during the experimental period. Daily administration was used in the acute DSS model to maintain continuous intestinal exposure during the period of active mucosal injury and rapid inflammatory progression. The OMV control group received purified ΔEC-derived OMVs by oral gavage at the indicated dose. Mice in all groups started receiving aTC (0.1 mg/ml) in drinking water to induce protein expression. Thus, all treatment comparisons were performed under the same aTC exposure baseline. DSS (2.5%; MP Biomedicals, Irvine, CA, USA) was added to the drinking water from days 0 to 7 to establish a colitis model. Following a 1-d recovery period, the mice were euthanized on day 8 for organ harvesting.

A chronic DSS-induced colitis model was utilized to further evaluate the therapeutic effect of the engineered probiotic over a 39-d period. While the grouping, dosing, treatment, and sampling protocols mirrored the acute model, the chronic model used 3 cycles of drinking water containing 2% DSS for 6 d, followed by normal water for 7 d. Administration of the engineered bacteria, purified IL-37b, and aTC was performed every other day throughout the study. In the chronic DSS model, administration was performed every other day to provide sustained but less intensive exposure during repeated injury–recovery cycles, while avoiding unnecessary cumulative treatment pressure over the extended disease course.

The in vivo safety of the engineered bacteria was evaluated in healthy female C57BL/6J mice. For short-term safety, mice received daily oral gavage of PBS, EcN, ΔEC, or ΔECIL-37b for 7 d. For repeated-dosing safety and immunogenicity assessment, mice received oral PBS, EcN, ΔEC, or ΔECIL-37b every other day for 28 d. Body weight, complete blood count, serum biochemistry, and major-organ histology were assessed. Serum anti-IL-37b antibody levels were measured by enzyme-linked immunosorbent assay using recombinant IL-37b-coated plates, and values of blank-subtracted optical density at 450 nm were calculated after serial dilution of serum samples. For fecal persistence analysis, fecal samples were collected at the indicated time points, homogenized, serially diluted, and plated on selective agar. Plasmid retention was assessed by comparing colony growth on selective and nonselective media during continuous passage. Horizontal transfer was evaluated by coincubating ΔECIL-37b with the indicated recipient strains and screening for transconjugants on appropriate selective media.

### H&E and periodic acid–Schiff staining

Distal colon tissues were fixed overnight in 4% paraformaldehyde. Fixed colon samples were embedded in paraffin and sectioned at 5 μm in thickness. H&E and periodic acid–Schiff staining were performed as previously described [49].

### Immunofluorescence and IHC

Paraffin sections of colon tissue were deparaffinized and antigen retrieval was performed with sodium citrate buffer. After rinsing in PBS, the sections were immersed in 0.1% Triton X-100 for 10 min at room temperature. The samples were blocked for 1 h using 3% bovine serum albumin and 10% donkey serum in PBS. Primary and secondary antibodies were added sequentially as appropriate (Table S1), and cell nuclei were stained using 4′,6-diamidino-2-phenylindole (DAPI; Beyotime, Beijing, China). In addition, 3,3′-diaminobenzidine (DAB) Substrate Kit (SK-4100, Vector, USA) and horseradish peroxidase (434323, Invitrogen, USA) were used for IHC. Tissue sections were sealed with an antifade mounting medium and imaged using a Leica TCS SP8 confocal microscope (Nussloch, Germany).

### qRT-PCR and flow cytometry

Colon tissues were processed for subsequent qRT-PCR and flow cytometry analyses. For qRT-PCR, tissue samples were homogenized in a bead-mill-type homogenizer at −40 °C prior to extraction of total RNA using an RNA extraction kit (Vazyme). Total RNA samples (1 μg) were reverse transcribed to cDNA using the QuantiTect Reverse Transcription Kit (Vazyme). qRT-PCR was performed using a Bio-Rad CFX instrument real-time PCR system (Hercules, CA, USA). The primer sequences are listed in Table S5. For flow cytometry, colon tissues were dissociated enzymatically at 37 °C for 1 h using a Dulbecco’s modified Eagle’s medium buffer containing murine collagenase type II (1 mg/ml; Solarbio), deoxyribonuclease I (0.01 mg/ml), and Dispase II (2 mg/ml; Sigma-Aldrich). The resulting cell suspension was filtered (70 μm), and immune cells were isolated via a 40%/80% Percoll gradient (Biosharp). Cells were then stained with surface markers (Table S1), and live cells were identified using Fixable Viability Stain 700 (BD Biosciences). Foxp3/Transcription Factor Staining Buffer Set (eBioscience, San Diego, CA, USA) was used for fixation, permeabilization, and intracellular staining as previously described [49]. Cells were analyzed using a flow cytometer (Beckman Coulter, Brea, CA, USA), and data were analyzed using FlowJo software provided with the instrument.

### Gut microbiota profiling

Fecal samples were collected at the end point of the chronic DSS experiment for 16S rRNA sequencing. Microbial DNA was extracted from fecal samples using the E.Z.N.A. Soil DNA Kit (Omega Bio-tek, USA) according to the manufacturer’s protocols. After passing quality control, the V3-V4 hypervariable region of the 16S rRNA gene was amplified by PCR with the 338F/806R primer set. The resulting amplicons were purified from agarose gels following the manufacturer’s instructions of the AxyPrep DNA Gel Extraction Kit (USA). Finally, paired-end sequencing was performed on an Illumina MiSeq platform by Microeco Tech Co. Ltd. (Shenzhen, China). The raw 16S rRNA gene sequencing data have been deposited in the National Center for Biotechnology Information Sequence Read Archive under BioProject accession number PRJNA1440636. β-Diversity differences were assessed by PERMANOVA. Genus-level pairwise comparisons were adjusted using Benjamini–Hochberg FDR correction.

### Western blot and Co-IP

Western blot experiments were performed as previously described [50]. Briefly, proteins were separated by 10% SDS–polyacrylamide gel electrophoresis, electrotransferred onto polyvinylidene difluoride membranes, and then incubated sequentially with primary and secondary antibodies using tris-buffered saline containing 0.5% Tween 20 (Table S1). Protein bands were visualized using enhanced chemiluminescence kit (Millipore, Burlington, MA, USA). For Co-IP, HEK293T cells were transfected with plasmids encoding IL-37b (46 to 218)-Flag, IL-18R-HA, and/or IL-1R8-His. At 24 to 48 h after transfection, cells were lysed with Co-IP lysis buffer containing protease inhibitors, and protein expression was verified by Western blotting. For OMV-IL-37b receptor-interaction analysis, ΔECIL-37b OMVs were incubated with lysates from IL-18R-HA- and IL-1R8-His-expressing HEK293T cells. The lysate mixtures were then incubated with target-specific antibodies and Protein A/G agarose beads (Magneti-G HA/His/IgG DYKDDDDK Tag Co-IP Kit, ACE Biotechnology, China) overnight at 4 °C. After washing, the immunoprecipitates were eluted with SDS loading buffer and analyzed by Western blotting.

### Statistical analysis

Data are presented as means ± SD or means ± SEM as indicated in the figure legends. Statistical analyses were performed using GraphPad Prism 8.0. Comparisons between 2 groups were analyzed by unpaired 2-tailed Student’s *t* test, and comparisons among multiple groups were analyzed by 1-way or 2-way analysis of variance (ANOVA) where appropriate. *P* < 0.05 was considered statistically significant.

## Ethical Approval

All animal procedures were approved by and conducted in accordance with the ethical regulations of the South China Agricultural University Animal Care Committee (approval numbers: 2022B119, 2022C116, 2025C100, 2025b011, and 2026c014).

## Data Availability

The raw 16*S* rRNA gene sequencing data generated in this study have been deposited in the National Center for Biotechnology Information Sequence Read Archive under BioProject accession number PRJNA1440636 and will be released upon publication. The source data underlying the quantitative results presented in the main and supplementary figures are provided in the accompanying Source Data file. Additional data supporting the findings of this study are available from the corresponding author upon reasonable request.
