# Supplementary material for: Programmed Probiotics Generate Interleukin-37b-Bearing Nanovesicles In Situ for Precision Immunomodulation in Colitis
Source: Research (Wash D C). 2026 Aug 28;9:1413. doi: 10.34133/research.1413 (PMC13522564; doi:10.34133/research.1413)
Supplement: Supplementary file 1 [file research.1413.f1.zip › Figs. S1 to S14 RESEARCH-D-26-02156.docx]

Supporting Information

**Programmed Probiotics Generate IL-37b-Bearing Nanovesicles in situ for Precision Immunomodulation in Colitis**

*Xiaojing Zhang, Liufei Gong, Haiyi Zhang, Yang Luo, Tengfei Long, Qian He, Yang Luo, Lu Han, Zhipeng Li, Cang Li, Xiaoyu Wang, Zijian Zheng, Yao Deng, Xiaoping Liao, Hao Ren^*^, Jian Sun^*^*

**Fig. S1.**

(A) Workflow for bacterial expression and purification of human IL-37b. The IL-37b gene fragment was commercially synthesized based on the human IL-37b sequence; (B) SDS-PAGE test for protein expression; (C) Western blot test for protein expression; (D) Relative expression of inflammation-related genes in mouse spleen; (E) Expression of IL-6 in treated RAW 264.7 cells; (F) Expression of IL-1β in treated RAW 264.7 cells; (G) Overview of experiments to validate the efficacy of IL-37b on primary intestinal cells; (H) Expression of IL-6 in treated primary intestinal cells; (I) Expression of IL-1β in treated primary intestinal cells. ns, not significant; **P* < 0.05; ****P* < 0.001.

**Fig. S2.**

(A) CRISPR/Cas9-mediated deletion of the *yrbE* gene; (B) Gel electrophoresis verification of *yrbE* gene deletion; (C) Diagram of engineered plasmid pTet-ClyA^M^-IL-37b; (D) Diagram of engineered plasmid pTet-ClyA^M^-GFP; (E) Diagram of engineered plasmid pTet-ClyA^M^-IL-37b-LuxCDABE.

**Fig. S3.**

(A) Growth curves of EcN, ΔE, ΔEC and ΔECIL-37b under standard culture conditions; (B) Growth curve of ΔECIL-37b after aTC induction compared with EcN, ΔE and ΔEC; (C–G) Growth curves of EcN and engineered derivatives under sodium deoxycholate stress at 0.025%, 0.05%, 0.1%, 0.2% and 0.3%, respectively; (H–L) Growth curves of EcN and engineered derivatives under pH 3, pH 4, pH 5, pH 6 and pH 7 conditions, respectively. Bacterial growth was recorded by OD600 measurement over 24 h. Data are presented as mean ± SD from three independent biological replicates.

**Fig. S4.**

(A) Western blot analysis of IL-37b in ΔECIL-37b OMVs after proteinase K digestion with or without EDTA treatment; (B) Western blot analysis and gray-level quantification of IL-37b loading in ΔECIL-37b OMVs using recombinant IL-37b as a reference; (C) Viability of Caco-2 cells treated with ΔECIL-37b OMVs at the indicated concentrations; (D) Hemolysis assay of ΔECIL-37b OMVs at the indicated concentrations. Triton X-100 was used as the positive control. Data are presented as mean ± SD.

**Fig. S5.**

(A) Representative ex vivo fluorescence images of major organs and gastrointestinal tissues collected at 3, 6, and 9 h after oral administration of DiR-labeled ΔECIL-37b OMVs or free DiR/PBS. (B–E) Quantification of normalized fluorescence intensity in the kidney (B), liver (C), spleen (D), and gastrointestinal tract (GI) (E) at the indicated time points. Fluorescence signals were measured using an IVIS imaging system, and the corresponding DiR/PBS fluorescence intensity was subtracted from the DiR-labeled ΔECIL-37b OMV signal at each time point for normalization. Data are presented as mean ± SD.

**Fig. S6.**

Relative expression of inflammation-related genes for the indicated experimental groups (A) TNF-α; (B) IL-6 and (C) IL-1β. Data are presented as mean ± SD. ns, not significant; **P* < 0.05; ***P* < 0.01; ****P* < 0.001; *****P* < 0.0001.

**Fig. S7.**

(A) Immunofluorescence images of MUC2 in different treatment groups; (B) Representative Western blots of ZO-1, Occludin, Claudin-1 and GAPDH; Quantification of (C) ZO-1, (D) Occludin and (E) Claudin-1 protein levels normalized to GAPDH (*n* = 8). (F) Flower (petal) plot of shared and unique OTUs across groups; (G) Shannon index of the gut microbiota in different groups; (H) Relative abundance of gut microbiota at the phylum level, shown as a percentage of total sequences. Relative abundance of (I) Firmicutes and (J) Bacteroidota from the feces of experimental mice. ns, not significant; **P* < 0.05; ***P* < 0.01; ****P* < 0.001; *****P* < 0.0001.

**Fig. S8.**

Plasmid maps of (A) HM617-IL1R8-His, (B) HM617-IL18Rα-HA, and (C) HM617-IL37b-Flag used for receptor-interaction analysis; (D) Co-immunoprecipitation analysis of the interaction among IL-37b-Flag, IL1R8-His and IL18R-HA using anti-Flag, anti-His or anti-HA beads. IgG was used as the negative control; (E–G) Relative mRNA expression levels of inflammatory cytokines, including TNF-α (E), IL-6 (F) and IL-1β (G), in water-treated control mice and DSS-treated mice receiving PBS, ΔECIL-37b, ΔEC or recombinant IL-37b. Data are presented as individual values with mean ± SEM. Statistical significance was determined by one-way ANOVA. ns, not significant; **P* < 0.05; ***P* < 0.01; ****P* < 0.001; *****P* < 0.0001.

**Fig. S9.**

(A) Representative Western blot images and quantification of MyD88 protein levels; (B) Representative MyD88 immunohistochemical staining images; (C) Quantification of MyD88 immunohistochemical staining. MyD88-positive areas were quantified using ImageJ. Data are presented as mean ± SD. **P* < 0.05; ***P* < 0.01; ****P* < 0.001.

**Fig. S10.**

(A) Schematic of the pharmacological validation workflow in RAW 264.7 cells; (B, C) RT-qPCR analysis of IL-6 and IL-1β under the ST2825 intervention setting; (D) Representative Western blots of p-ERK, ERK, p-p65, p65, and GAPDH under the ST2825 intervention setting; (E, F) Densitometric quantification of p-ERK/ERK and p-p65/p65 ratios from panel D; (G, H) RT-qPCR analysis of IL-6 and IL-1β under the PMA intervention setting; (I) Representative Western blots of p-ERK, ERK, p-p65, p65, and GAPDH under the PMA intervention setting; (J, K) Densitometric quantification of p-ERK/ERK and p-p65/p65 ratios from panel I. Data are presented as mean ± SD. **P* < 0.05; ***P* < 0.01; ****P* < 0.001; *****P* < 0.0001.

**Fig. S11.**

(A) Flow cytometry gating strategy for CD45⁺ immune cell populations; (B) Effect of treatment on dendritic cell populations in the lamina propria of colitis mice.

**Fig. S12.**

(A) Schematic diagram for safety evaluation of animal experiments; (B–I) Complete blood count parameters measured following blood collection on day 8; (J–M) Serum biochemical parameters associated with liver and kidney function. ALT: alanine aminotransferase; AST: aspartate aminotransferase; BUN: blood urea nitrogen; CREA: creatinine; (N) Representative H&E staining images of the major organs of mice treated with PBS, EcN, ΔEC and ΔECIL-37b. Scale bar, 100 μm. Data are presented as mean ± SD.

**Fig. S13.**

(A) Schematic illustration of the 28-day repeated administration protocol with PBS, EcN, ΔEC or ΔECIL-37b; (B) Body weight changes during the treatment period; (C) Routine blood parameters, including white blood cells, red blood cells, hematocrit, mean corpuscular volume, mean corpuscular hemoglobin, mean corpuscular hemoglobin concentration, hemoglobin and platelets; (D) Serum biochemical indicators, including ALT, AST, BUN and CREA; (E) Representative H&E staining images of major organs, including heart, liver, spleen, lung and kidney. Scale bars, 100 μm. Data are presented as mean ± SD.

**Fig. S14.**

(A) Serum anti-IL-37b antibody levels in mice treated with ΔEC or ΔECIL-37b, determined by IL-37b-coated ELISA and shown as blank-subtracted OD450 values at different serum dilutions; (B) Fecal abundance of ΔECIL-37b after administration, expressed as CFU/g feces. LOD, limit of detection; (C) Plasmid retention of ΔEC and ΔECIL-37b during continuous passage; (D) Conjugation frequency of the engineered plasmid from ΔECIL-37b to recipient strains, including *E. coli* C600, *Salmonella* 15 and *K. pneumoniae* 103. ND, not detected. Data are presented as mean ± SD.
