# Supplementary material for: Programmed Probiotics Generate Interleukin-37b-Bearing Nanovesicles In Situ for Precision Immunomodulation in Colitis
Source: Research (Wash D C). 2026 Aug 28;9:1413. doi: 10.34133/research.1413 (PMC13522564; doi:10.34133/research.1413)
Supplement: Supplementary file 1 [file research.1413.f1.zip › Table S1 to S8 RESEARCH-D-26-02156.docx]

Supporting Information

**Programmed Probiotics Generate IL-37b-Bearing Nanovesicles in situ for Precision Immunomodulation in Colitis**

*Xiaojing Zhang, Liufei Gong, Haiyi Zhang, Yang Luo, Tengfei Long, Qian He, Yang Luo, Lu Han, Zhipeng Li, Cang Li, Xiaoyu Wang, Zijian Zheng, Yao Deng, Xiaoping Liao, Hao Ren^*^, Jian Sun^*^*

**Table S1. Summary of the various types of antibodies.**

| **Antibody** | **Dilution** | **Application** |
| --- | --- | --- |
| Anti-IL-37b antibody (Abcam, USA) | 1:1000 | Western blot |
| Anti-ZO1 antibody (ThermoFisher, USA) | 1:2000 |  |
| Anti-Occludin antibody (ABclonal, Wuhan, China) | 1:1000 |  |
| Anti-Claudin-1 antibody (Bioss, Beijing, China) | 1:1000 |  |
| Anti-GAPDH antibody (Affinity, Shanghai, China) | 1:1000 |  |
| Anti-MyD88 antibody (Bioss, Beijing, China) | 1:1000 |  |
| Anti-p44/42 MAPK (ERK1/2) antibody (CST, USA) | 1:1000 |  |
| Anti-Phospho-p44/42 MAPK (ERK1/2) antibody (CST, USA) | 1:1000 |  |
| Anti-Phospho-NF-κB p65 antibody (CST, USA) | 1:1000 |  |
| Anti-NF-κB p65 antibody (CST, USA) | 1:1000 |  |
| Anti-iNOS antibody (CST, USA) | 1:1000 |  |
| Anti-COX-2 antibody (CST, USA) | 1:1000 |  |
| Goat Anti-Rabbit/Mouse IgG Antibody (H+L), HRP (EpiZyme, Shanghai, China) | 1:5000 |  |
| Anti-MUC2 antibody (Abcam, USA) | 1:200 | Immunofluorescence |
| Alexa Fluor 488, donkey anti-rabbit (Invitrogen Molecular Probes, USA) | 1:500 |  |
| Anti-MyD88 antibody (Bioss, Beijing, China) | 1:200 | Immunohistochemistry |
| Anti-MPO antibody (HUABIO, China) | 1:500 |  |
| Anti-CD45-APC-Cy7 (Biolegend, USA) | 1:200 | Flow Cytometry |
| Anti-CD11b-PE-Cy7 (Biolegend, USA) | 1:200 |  |
| Anti-Ly6G-PerCP-Cy5.5 (Biolegend, USA) | 1:200 |  |
| Anti-F4/80-APC (Biolegend, USA) | 1:200 |  |
| Anti-CD86-BV421 (Biolegend, USA) | 1:200 |  |
| Anti-CD206-PE (Biolegend, USA) | 1:200 |  |
| Anti-CD11c-PE (Biolegend, USA) | 1:200 |  |
| Anti-MHCII-V500 (Biolegend, USA) | 1:200 |  |
| Fixable viability stain 700 (Biolegend, USA) | 1:200 |  |

**Table S2. Strains and plasmids used in this study.**

| **Strain abbreviation** | **Genomic modification/plasmid** | **Comment** |
| --- | --- | --- |
| *E. coli* BL21 (DE3) | / | Host strain for recombinant protein expression |
| */* | pET28a-IL-37b | Plasmids for specific expression of IL-37b |
| EcN | / | *E. coli* Nissle 1917 (GenBank: CP007799.1) |
| EcN/pCaskp | pCaskp | EcN with Cas9 protein |
| / | pSGkp-*yrbE* (gRNA plasmid) | Engineered plasmids targeting the *yrbE* gene |
| ΔE | Δ*yrbE* | Hypervesiculating EcN |
| ΔEC | pTet-ClyA^M^ | Hypervesiculating EcN not expressing IL-37b protein, used as a control in animal experiments. |
| ΔECIL-37b | pTet-ClyA^M^-IL-37b | Hypervesiculating EcN expressing membrane-confined IL-37b |
| ΔEC-GFP | pTet-ClyA^M^-GFP | This strain was used to extract GFP-containing OMVs to study subsequent cellular uptake |
| ΔEC-Lux | pTet-ClyA^M^-IL-37b-LuxCDABE | Hypervesiculating EcN with luciferase for protein expression *in vivo* study |
|  | pET28a-J23119-ClyA^M^-IL-37b | Constitutive expression of IL-37b protein |

**Table S3. Primers used to knock out the *yrbE* gene.**

| **Forward primers** | **Reverse primers** | **Comments** |
| --- | --- | --- |
| TAGTCAACTGTCTGATTAAGAGCGGTTTTAGAGTAGAAATAGCAAG | AAACCGCTCTTAATCAGACAGTTGACTGGTATTATACCTAGGCTGAG | the 20 bp spacer sequence targeting *yrbE* |
| CAACTGTCTGATTAAGAGCG | GCCGCTCTAGAAGTAGTGGA | Verify plasmid gRNA substitution |
| GTATCGAATTCCTGCAGCCCGGGCTGTATACAGTGCGCA | TTCATTTTTTTTCGTTTGCATGAACGAGTGGCTTAACTCC | Amplification of the upstream homology arm of the *yrbE* gene |
| GTTCATGCAAACGAAAAAAAATG | AGAAGTAGTGGATCCCCCGGGTTAAACATGCGTCGGTTC | Amplification of the homology arm downstream of the *yrbE* gene |
| TCTCGTTTGGATTGCAACTG | GCCGCTCTAGAAGTAGTGGA | Test for homologous arm substitution |
| GGTCGGTTCAGTACGTATGG | TGTCGCTCATGGCAGTGC | Test for deletion of the *yrbE* gene |

**Table S4. Primers used to construct the engineered plasmids.**

| **Forward primers** | **Reverse primers** | **Comments** | **Goal** |
| --- | --- | --- | --- |
| ATCGCGGATCCGTTCACACAAGTCCAAAGGTG | ACCCATTTGCTGTCCACC | Addition of homology arms and reverse amplification of pET28a-IL-37b plasmid | Constructing the pTet-ClyA^M^-IL-37b plasmid |
| CATGACTGGTGGACAGCAAATGGGTATGACTGAAATCGTTGCA | ACCTTTGGAGAAGTATGAACAGAACCACCACCACCAGAACCACCACCACCTGAACCACCACCACCGACTTCAGGTACCTC | Amplification of ClyA sequence (*E. coli* MG1655) |  |
| TGCCGCAGATGATGTCGTCGCCGGTC | CGACGACATCATCTGCGGCACCGGC | Sites 182–184 of the mutant ClyA sequence |  |
| TGTCCTACGAGTTGCATGATAAAGA | TCATAGATCCTTTCTCCTCTTTAGAT | Replacement promoter for tetR/O |  |
| CGTCCGGCGTAGAGGATCGAGATC | ATCCGGATATAGTTCCTCCTTTC | Detection of pTet-ClyA^M^-IL-37b plasmid construction completion |  |
| CGCGGATCCGAATTCGAG | AGCTCGAATTCGGATCCGCGGACTTCAGGTACCTCAAAGAGTGTC | Deletion of IL-37b sequence | Constructing the pTet-ClyA^M^ plasmid |
| CGCGGATCCGAATTCGAGCTCCGTCGACAA | AGAACCACCACCACCAGAACCACCACCACC | Reverse amplification pTet-ClyA^M^ plasmid | Constructing the pTet-ClyA^M^-GFP plasmid |
| TGGTGGTTCTGGTGGTGGTGGTTCTATGCGTAAAGGCGAAGAGCTGTTCACT | GACGGAGCTCGAATTCGGATCCGCGTTATCATCATTTGTACAGTTCATCCATACCATGCGT | Amplification of GFP (GeneBank Accession No. HQ873313.1) |  |
| CGCGGATCCGAATTCGAG | AGAACCACCACCACCAGAACCACCACCACCTGAACCACCACCACCATCGCTGACCTCACTGGG | Reverse amplification of pTet-ClyA^M^-IL-37b plasmids and addition of linker sequences | Constructing the pTet-ClyA^M^-IL-37b-LuxCDABE plasmid |
| GGTGGTGGTGGTTCAGGTGGTGGTGGTTCTGGTGGTGGTGGTTCTATGACTAAAAAAATTTCATTCATTATTAACGGC | CTCGAATTCGGATCCGCGGCAAGCATTCCACTTACAATTAGG | Amplification of LuxCDABE sequence and addition of linker sequence |  |

**Table S5. Primers used for qRT-PCR.**

| **Forward primers** | **Reverse primers** | **Gene** |
| --- | --- | --- |
| CCCTCACACTCAGATCATCTTCT | GCTACGACGTGGGCTACAG | TNF-α |
| CTGCAAGATACTTCATCCAG | AGTGGTATAGACAGGTCTGTTGG | IL-6 |
| GCAACTGTTCCTGAACTCAACT | ATCTTTTGGGGTCCGTCAACT | IL-1β |
| GCTCTTACTGACTGGCATGAG | CGCAGCTCTAGGAGCATGTG | IL-10 |
| CCGCTCGCTTCTCTGTGC | CTCTGGATGTTCTTGAGGTGAATC | CXCL-1 |
| CCAACCACCAGGCTACAG | CTTCAGGGTCAAGGCAAAC | CXCL-2 |
| TTAAAAACCTGGATCGGAACCAA | GCATTAGCTTCAGATTTACGGGT | MCP-1 |
| TGCGTGACATCAAAGAGAAG | TCCATACCCAAGAAGGAAGG | β-actin |

**Table S6. Histological grading scheme for DSS colitis.**

| **Parameter graded** | **Score** | **Description** |
| --- | --- | --- |
| Inflammation | 0 | None |
|  | 1 | Slight |
|  | 2 | Severe |
| Depth of injury | 0 | None |
|  | 1 | Mucosa |
|  | 2 | Mucosa and submucosa |
|  | 3 | Transmural |
| Crypt damage | 0 | None |
|  | 1 | Basal one-third damaged |
|  | 2 | Basal two-thirds damaged |
|  | 3 | Only surface epithelium intact |
|  | 4 | Entire crypt and epithelium lost |
| Percent involvement | 1 | 1–25% |
|  | 2 | 26–50% |
|  | 3 | 51–75% |
|  | 4 | 76–100% |

**Table S7.** **DAI scoring scheme for DSS colitis.**

| **Parameter graded** | **Score** | **Description** |
| --- | --- | --- |
| Weight loss (%) | 0 | < 1% |
|  | 1 | 1–6% |
|  | 2 | 6–12% |
|  | 3 | 12–18% |
|  | 4 | > 18% |
| Diarrhea severity | 0 | Normal |
|  | 1 | Loose stool but still formed |
|  | 2 | Loose stool |
|  | 3 | Very loose and wet stool |
|  | 4 | Watery diarrhea |
| Rectal bleeding | 0 | Normal |
|  | 1 | Brown |
|  | 2 | Red |
|  | 3 | Blood traces visible in stool |
|  | 4 | Gross rectal bleeding |
| DAI = (weight loss score + diarrhea score + rectal bleeding score) / 3 | | |

**Table S8. Pairwise PERMANOVA analysis of gut microbiota β-diversity based on Bray–Curtis distance.**

| **Group 1** | **Group 2** | **pseudo-F** | **P value** | **q value** |
| --- | --- | --- | --- | --- |
| CON | DSS | 12.5689 | 0.001 | 0.00375 |
| CON | ΔEC | 14.6290 | 0.002 | 0.005 |
| CON | ΔECIL-37b | 14.3667 | 0.001 | 0.00375 |
| CON | IL-37b | 4.4528 | 0.002 | 0.005 |
| CON | OMV | 11.8686 | 0.001 | 0.00375 |
| DSS | ΔEC | 1.9335 | 0.049 | 0.05769 |
| DSS | ΔECIL-37b | 3.0229 | 0.001 | 0.00375 |
| DSS | IL-37b | 2.5369 | 0.013 | 0.0195 |
| DSS | OMV | 0.3886 | 0.939 | 0.939 |
| ΔEC | ΔECIL-37b | 2.0854 | 0.018 | 0.02455 |
| ΔEC | IL-37b | 3.0263 | 0.006 | 0.01125 |
| ΔEC | OMV | 1.5435 | 0.149 | 0.15964 |
| ΔECIL-37b | IL-37b | 2.6042 | 0.013 | 0.0195 |
| ΔECIL-37b | OMV | 2.7503 | 0.004 | 0.00857 |
| IL-37b | OMV | 2.0344 | 0.050 | 0.05769 |
